# Capture Hi-C identifies the chromatin interactome of colorectal cancer risk loci

**DOI:** 10.1038/ncomms7178

**Published:** 2015-02-19

**Authors:** Roland Jäger, Gabriele Migliorini, Marc Henrion, Radhika Kandaswamy, Helen E. Speedy, Andreas Heindl, Nicola Whiffin, Maria J. Carnicer, Laura Broome, Nicola Dryden, Takashi Nagano, Stefan Schoenfelder, Martin Enge, Yinyin Yuan, Jussi Taipale, Peter Fraser, Olivia Fletcher, Richard S. Houlston

**Affiliations:** 1 Division of Genetics and Epidemiology, The Institute of Cancer Research, Sutton, Surrey SM2 5NG, UK; 2 Division of Molecular Pathology, The Institute of Cancer Research, Sutton, Surrey SM2 5NG, UK; 3 Division of Molecular Pathology, Haemato-Oncology Research Unit, The Institute of Cancer Research, Sutton, Surrey SM2 5NG, UK; 4 Breakthrough Breast Cancer Research Centre, The Institute of Cancer Research, London SW3 6JB, UK; 5 Nuclear Dynamics Programme, The Babraham Institute, Cambridge CB22 3AT, UK; 6 Department of Biosciences and Nutrition, Science for Life Laboratory, Karolinska Institutet, 14 183, Huddinge, Sweden

## Abstract

Multiple regulatory elements distant from their targets on the linear genome can influence the expression of a single gene through chromatin looping. Chromosome conformation capture implemented in Hi-C allows for genome-wide agnostic characterization of chromatin contacts. However, detection of functional enhancer–promoter interactions is precluded by its effective resolution that is determined by both restriction fragmentation and sensitivity of the experiment. Here we develop a capture Hi-C (cHi-C) approach to allow an agnostic characterization of these physical interactions on a genome-wide scale. Single-nucleotide polymorphisms associated with complex diseases often reside within regulatory elements and exert effects through long-range regulation of gene expression. Applying this cHi-C approach to 14 colorectal cancer risk loci allows us to identify key long-range chromatin interactions in *cis* and *trans* involving these loci.

## Abstract (web summary)

Multiple regulatory elements distant from their targets on the linear genome can influence gene expression through chromatin looping. Here, the authors report an improved chromosome conformation capture approach that can be used to identify long-range chromatin interactions in cancer risk loci.

## Graphical abstract

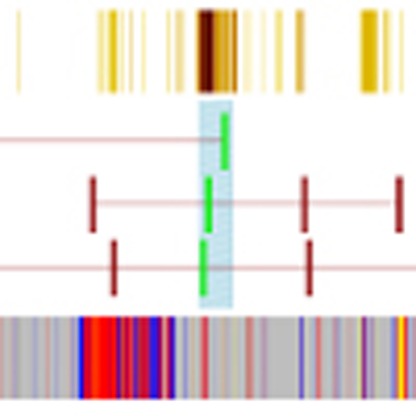

It is now recognized that the expression of a single gene is often influenced by multiple regulatory elements that can be kilobases (kb) to megabases (Mb) upstream or downstream of their targets^1^. Physical interactions between enhancers and promoters can be identified by chromosome conformation capture (3C^2^)-based methods, which are performed through the digestion and re-ligation of fixated chromatin followed by enumeration of ligation junctions^3^. While not all physical interactions are regulatory, in addition to *cis*-regulation there is evidence for *trans-*interaction^4^,^5^,^6^,^7^, which may be functional. Although powerful, only interactions that have been considered *a priori* can be detected using 3C. Extensions to 3C (for example, 4C^4^) allow for the sampling of all possible interactions with a constant fragment. When multiple intra- and inter-chromosomal targets are possible, an agnostic method of detection is required. Although Hi-C^7^ enables the detection of long-range interactions on a genome-wide scale, its effective resolution, which is contingent on restriction fragments and experimental sensitivity, prohibits the characterization of specific interactions.

Genome-wide association studies (GWASs) have identified single-nucleotide polymorphisms (SNPs) that are associated with complex diseases. As far as they have been deciphered these SNPs reside within regulatory elements and exert effects through long-range regulation of gene expression^8^,^9^,^10^.

Here we report a novel enhancement of Hi-C using target sequence enrichment, capture Hi-C (cHi-C), allowing for state of the art characterization of chromatin interactomes. We apply cHi-C to 14 colorectal cancer (CRC) risk loci^11^,^12^,^13^,^14^,^15^,^16^,^17^ to identify key long-range chromatin interactions involving these regions.

## Results

### Analysis of cHi-C data

The coverage of Hi-C was increased by enriching for specific genomic regions using RNA baits—providing for enrichment in excess of 130-fold (Supplementary Table 1). Local structures within the genome (fragment length bias, GC content and mapability), as well as unequal distribution of restriction sites can bias contact frequency^7^. To adjust for this, we normalized data on the principle of overall genome-wide interactivity adapted for cHi-C (Supplementary Equation 1; Supplementary Fig. 1). Since contact probability decreases with distance, the interaction frequency of any pair of intra-chromosomal loci was distance normalized (Supplementary Fig. 2). Hi-C contacts represent an ensemble average of functional, steric and random chromatin interactions. To identify relevant interactions, we analysed the underlying distribution of events, testing for significance.

### Application of cHi-C to CRC risk loci

To apply cHi-C to the 1q41, 3q26.2, 8q23.1, 8q24.21, 10p14, 11q23, 12q13, 14q22.2, 15q13, 16q22.1, 18q21.1, 19p13.1, 20p12.3 and 20q13.33 CRC risk loci^11^,^12^,^13^,^14^,^15^,^16^,^17^, we first refined the association signals. We used meta-data from analysis of five GWAS of CRC^18^. At each risk locus we defined our regions to include all SNPs with minor allele frequencies of 2% or greater, and that were correlated (*r*^2^>0.2) with the published SNP (Supplementary Table 2). We excluded rare SNPs and used *r*^2^ (rather than D′) as the metric for linkage disequilibrium, since GWAS are predicated on the assumption that the arrayed SNPs have a reasonably high correlation with common causal variants and while rare causal variants are also possible, they are less likely^19^,^20^,^21^.

A total of 4.68 Mb comprising these regions was subjected to cHi-C in libraries generated in LS174T, LoVo and Colo205 CRC cell lines. Inherent to the cHi-C method, interactions with the captured regions were generated at increased coverage on a genome-wide scale. For each cell line, next-generation sequencing (NGS) reads comprising two fragments (Hi-C ditags, one fragment each from the captured region and its ligated interacting partner, respectively) were uniquely mapped to equally sized genomic blocks (9 and 3 kb bins) and assigned to a genome-wide enrichment contact matrix (Supplementary Fig. 3).

We derived the chromatin interactome for the 14 risk loci, defined as genome-wide generic chromatin interactions significant at a 5% false discovery rate (FDR), in all of the three CRC cell lines at 9 kb resolution (parametric test using a zero-inflated Weibull distribution; Supplementary Data 1). These generic contacts were observed at a significantly higher frequency to that expected (*P*<10^−16^, combinatorial test). Statistically significant long-range interactions (>10 kb) were shown at all 14 risk loci. These contacts were not restricted to adjacent genes.

Transcription factor (TF)-binding-mediated chromatin interactions can be detected within a certain interval of the actual core TF-binding site (TFBS), depending on clusters formed by the specific TF^22^. Therefore, we complemented the genome-wide 9-kb analysis (Supplementary Data 1) with an analysis at 3 kb resolution in close-*cis* (+/−5 Mb of the risk linkage disequilibrium (LD) block), revealing refined chromatin interactions for nine of the 14 CRC risk loci (Supplementary Data 2).

### Interactions at specific risk loci

**Figure 1:**
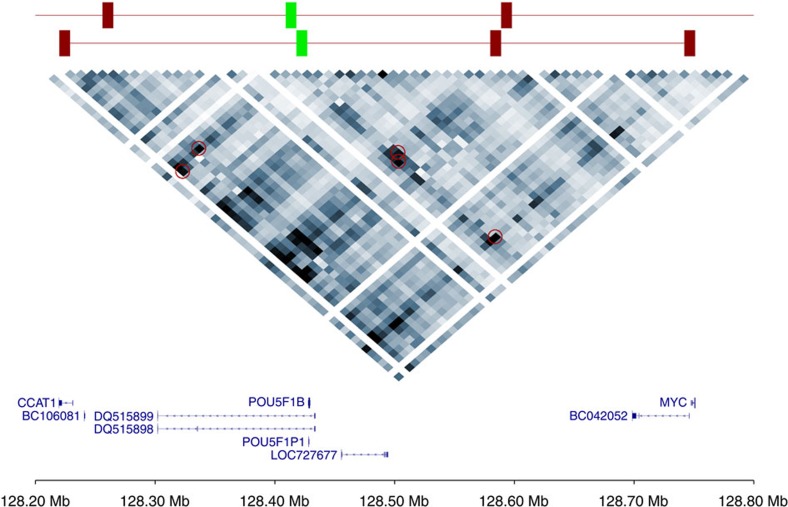
Heatmap of the 9-kb interaction matrix for the 8q24.21 capture region. Upper track shows five significant interactions (red) with two of the test bins (green). Heatmap intensity values represent an average of the data from the three cell lines.

**Figure 2:**
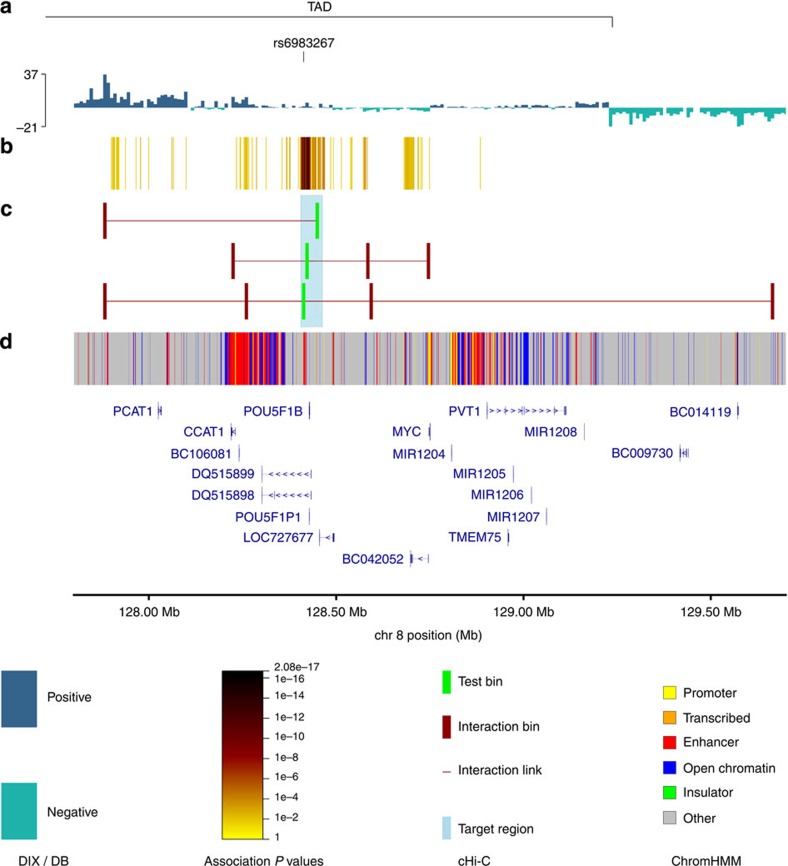
Integrative annotation of the 8q24 risk locus. (**a**) Topologically associating domain (TAD) borders at 8q24.21 consistently observed in LS174T, LoVo and Colo205 cell lines, as determined by domain calling on the directionality index (DIX). Positive and negative values indicate preferential downstream and upstream interactivity of a bin, respectively. (**b**) Statistical significance of the CRC association across the region; the darker the colour the stronger the association. The top associated SNP in the region is rs6983267. (**c**) Statistically significant looping interactions. Test bins in green, interactor bins in red. (**d**) Regulatory elements in HCT116.

To date, the most extensively studied cancer risk locus is at 8q24.21 (rs6983267)^8^,^9^,^23^. Figure 1 shows a heatmap for the enriched region encompassing this risk locus with significant interactions highlighted. The majority of interactions observed at 8q24.21 and in particular the known interaction between rs6983267 and *MYC* (Fig. 2), occur within a single chromatin interaction domain. Chromatin interactions have been shown to segregate into Mb-sized local chromatin domains, so-called topologically associating domains (TADs)^24^,^25^. Interestingly, the TAD we identified at 8q24.21 in colonic tissue (Fig. 2) overlays with and refines the corresponding TAD boundaries identified by Dixon *et al.*^25^ in human cell lines IMR90 and hESC (hg19/chr8:127880000–128800000 and hg19/chr8:127920000–130840000), thus implying generic regulatory function. At 8q24.21, we observed regulatory interactions at an increased frequency within TADs. Specifically, in addition to confirming the interaction between rs6983267 and *MYC*, we identified the MYC-regulated long non-coding RNA (lncRNA) *CCAT1* as an upstream interactor (Fig. 2). From cHi-C analysis of non-CRC cell lines SUM44, GM12878 and IMR90 cell lines for this 8q24.21 region, in contrast to the generic interaction upstream of *PCAT1*, the interaction peak at *CCAT1* was not apparent in IMR90 and GM12878 (Supplementary Fig. 4). These observations are concordant with recent data from Xiang *et al.*^26^ showing the role of *CCAT1-L*, a CRC-specific isoform of the *CCAT1* lncRNA, in intra-chromosomal looping with the *MYC* gene promoter regulating *MYC* transcription.

**Figure 3:**
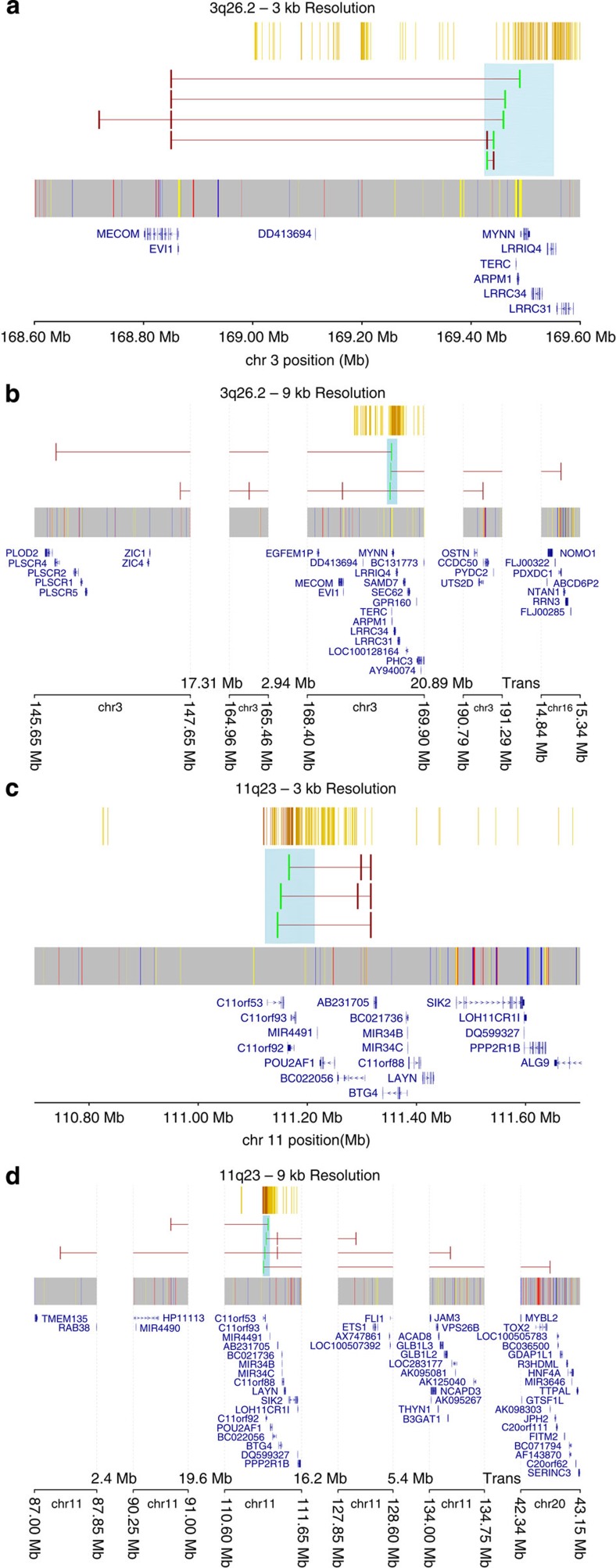
Annotation of significant chromatin interactions at 3q26.2 and 11q23. A genome-wide analysis at 9 kb resolution of 3q26.2 (**b**) and 11q23 (**d**) and a close-*cis-* (±5 Mb) analysis at 3 kb resolution of the same two regions (**a**,**c**) were performed.

At 3q26.2, several elements significantly interact with *MECOM*, a transcriptional regulator and oncoprotein affecting transforming growth factor-β signalling in CRC^27^ (Fig. 3). At 11q23, interactions with a region encoding the uncharacterized protein AB231705 were consistently seen at 3 and 9 kb resolution (Fig. 3). Genome-wide analysis of the 11q23 locus revealed both far-*cis-* and *trans-*interactions mapping close to the ETS1 oncogene^28^ (Fig. 3). Two different sets of looping interactions were seen at 14q22.2, consistent with genetic association data, suggesting the existence of two independent risk loci^17^ (Supplementary Figs 5–22). The high chromatin interactivity at 14q22.2 reflects a high density of promoters and enhancers in this region. For the 15q13, 18q21.1 and the 20p12.3 loci, the interaction network identifies a target gene proximal to the risk variant and regulated by distal elements. For others, conversely, the network suggests risk loci to participate in far-*cis-* and *trans-*regulation of genes implicated in cancer, including *TRPS1* (8q23)^29^, *TPO* (10p14)^30^, *VEZT* (12q13)^31^ and *RAN* (12q13)^32^, all recurrently mutated in CRC^33^,^34^ (Supplementary Figs 5–22).

**Figure 4:**
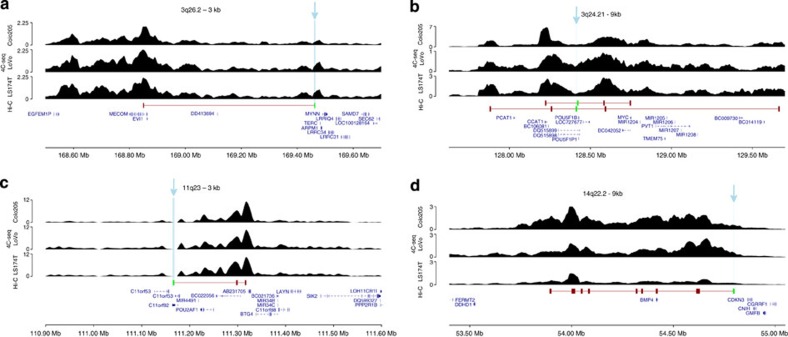
4C-seq analysis of cHi-C contacts at different genomic regions. The top three tracks of each panel show 4C-seq interactions for Colo205, LoVo and LS174T cell lines with significant Hi-C contacts overlapping the 4C-seq viewpoint on the fourth track. Genes and transcripts mapping to respective regions are also shown. The 4C-seq viewpoints have been indicated as light-blue-shaded boxes and with arrows pointing at them. (**a**) 3q26.2, (**b**) 8q24.21, (**c**) 11q23 and (**d**) 14q22.2.

**Figure 5:**
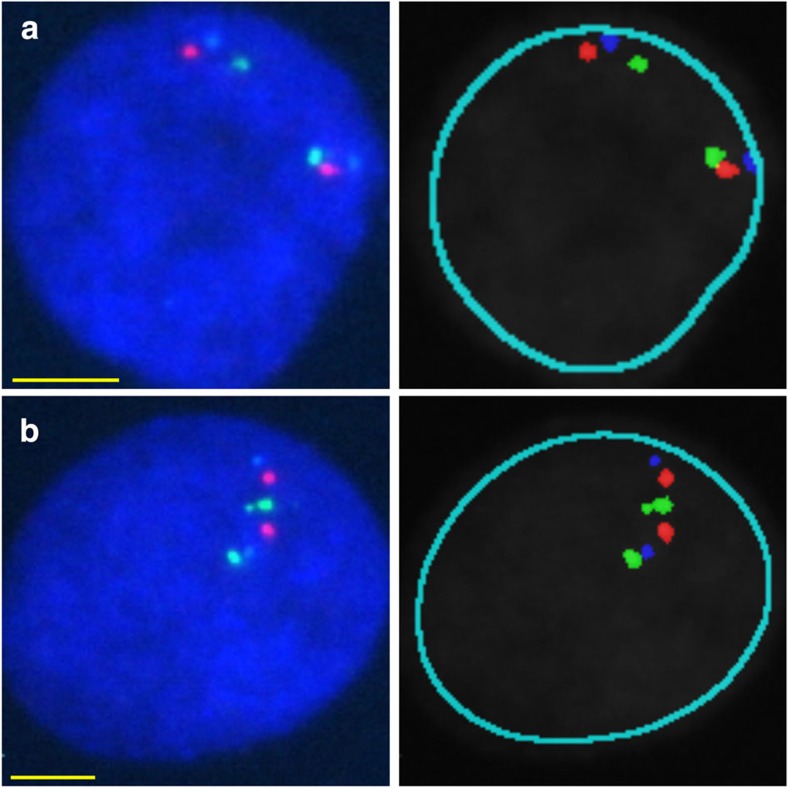
Analysis of co-localization frequencies at cHi-C contacts using interphase FISH. Three-colour probe sets were designed to cover cHi-C test bins (green), far-*cis*-interactor bins (red) and control elements (blue), the latter picked randomly at ~5 Mb distance from the interactor bin (Supplementary Methods; Supplementary Table 3). Shown are representative raw (left) and *in silico* processed (right) images from probe set cis_5 (Supplementary Table 3) applied onto interphase nuclei of LS174T cells (scale bars, 5 μm), confirming the significant cHi-C interaction at the 18q21 risk locus (9kb_contact_135; Supplementary Data 1). (**a**) Co-localization of the test bin with the cHi-C interactor bin. (**b**) Co-localization of the test bin with the control element. Co-localization of cHi-C interactions as shown in **a** was observed at significantly higher frequency compared with random background co-localization as shown in **b** (Supplementary Table 3).

To validate cHi-C results for 3q26.2, 8q24.21, 11q23 and 14q22.2, we used 4C-seq to examine close-*cis-*interactions (20 interactions, four viewpoints overlapping significant cHi-C contacts) in CoLo205, LoVo and LS174T cell lines. In all cases, significant cHi-C contacts were reflected consistently in the 4C-seq profiles (Fig. 4). In addition we designed fluorescence *in situ* hybridization (FISH) probes to validate seven far-*cis-* (>5 Mb) and seven *trans-*cHi-C interactions (Supplementary Table 3; Fig. 5) as an orthogonal methodology^4^. Seven of the assays were informative in terms of probe specificity and minimal number of counts required to establish a statistically significant relationship between probe co-localization (Supplementary Table 3). For these, co-localization frequencies were all significantly higher than background frequencies (*P*<0.05 Fisher’s exact test; also significant after FDR correction; Supplementary Table 3).

### Contacts are enriched at functional motifs

We integrated the CRC risk interactome with association fine-mapping and epigenetic profiling data defined by ChromHMM (Methods). Within GWAS signals, chromatin interactions were significantly enriched at sites overlaying the strongest SNP association (*P*=8.54 × 10^−3^ Fisher’s exact test; Supplementary Table 4). cHi-C contacts were enriched for regulatory elements (both enhancers and promoters: *P*<10^−7^ Fisher’s exact test), and this enrichment showed evidence for being tissue specific (Methods; Supplementary Table 6). Functional chromatin interactions have been proposed to arise from specific TF binding^3^,^35^. Integrating cHi-C data with CRC-specific TFBSs identified by chromatin immunoprecipitation (ChIP)-Seq (433 TFs in LoVo cells), chromatin interactions were significantly enriched for shared TFs (*P*=1.14 × 10^−14^ Fisher’s exact test; Supplementary Fig. 23). *A priori* this may be reflective of an increased specific TF binding at functional variants. While not universal, there is evidence that evolutionary conservation can be indicative of regulatory elements^36^. It was therefore possible to delineate SNPs with evidence for being causative on the basis of their profiles for evolutionary conservation, TF binding and chromatin state (Methods; Supplementary Table 5).

## Discussion

To increase the effective resolution of conventional Hi-C by a factor of *n* requires *n*^2^ sequencing reads, which is therefore prohibitive for general implementation. In contrast, the target sequence capture approach described herein allows for increasing the effective resolution in an approximately linear fashion, and therefore represents a far more cost-effective approach to identifying important chromatin interactions. HindIII restriction sites are located in the human genome at an average of 3 kb apart, limiting the intrinsic resolution that can be achieved. Experimentally, the effective resolution of cHi-C is also dictated by the coverage, which impacts both on the normalization and the statistical modelling procedure. The iterative bias normalization procedure, adapted from the iterative correction and eigenvector decomposition (ICE) protocol^37^, applied to our data set was shown to converge at the intrinsic 3 kb resolution for close-*cis* (<5 Mb) and at a three times lower resolution (9 kb) for genome-wide contacts, respectively (Supplementary Fig. 24). Furthermore, we were able to fit a parametric distribution to the resulting normalized reads (that is, convergence to a stable maximum of the likelihood optimization; Supplementary Fig. 25).

In our application of cHi-C to examine the CRC risk loci, in addition to identifying long-range *cis-*interactions our observations provide evidence to support the previously reported existence of *trans-*interactions^4^,^5^,^6^,^7^. While such inter-chromosomal interactions can be predicted from nuclear organization, it remains to be established whether they have direct functional significance.

The risk loci identified by GWAS are providing novel insights into disease biology. Compared with the great number of risk loci identified, the functional basis of only a limited number have, however, been elucidated to date. Where a GWAS association signal can be unambiguously assigned to a single SNP or can be defined by a restricted set of SNPs mapping to a small genomic region, 4C-seq^38^ undoubtedly provides a powerful method for the sampling of all possible interactions with such a constant fragment. However, in most cases the regions of association are not so well defined requiring multiple interrogations. The introduction of cHi-C provides an agnostic means of rapidly exploring many large genomic regions as viewpoints in contrast to 4C-seq. Contemporaneously with our cHi-C methodology has been the introduction of Capture-C^39^, which allows for the identification of close-*cis-*regulatory elements for a number of regions in a single experiment. Capture-C may afford better resolution to cHi-C, as it is based on a four-base pair (bp) restriction cutter, as compared with our current implementation of cHi-C, which has been based on a six-bp cutter. While there are no filtering statistics so far for Capture-C, the absence of a biotin pull-down may have the consequence that cHi-C may offer a superior signal to noise ratio compared with Capture-C, cHi-C addressing non-ditag fragment background contamination.

Here our analysis has been predicated on observing significant interactions in all three CRC cell lines subjected to cHi-C. While this has afforded an opportunity to reveal generic interactions that play a role in divergent aetiologies, we acknowledge that it is likely that cell-specific interactions will exist in CRC; additionally, some may be specific to cell lines rather than CRC *per se*.

Our analysis has revealed a complex interaction network for most of the risk loci often implicating bi-directional regulation, as well as long-range interactions. While these remain to be elucidated, we were able to confirm documented interactions and reveal novel interactions between these and plausible biological candidates, thus extending knowledge of salient networks. At 8q24, in addition to confirming the interaction between rs6983267 and *MYC*, we identified *CCAT1* as an upstream interactor. *CCAT1* is upregulated in CRC^40^,^41^ and intriguingly *CCAT2*, another lncRNA that is encoded by the rs6983267 locus, is a regulator of *MYC* and a Wnt target^42^. Collectively, these data suggest a regulatory network involving looping interactions between *CCAT2*, *CCAT1* and *MYC*, as well as Wnt-feedback regulation.

Similarly, the cHi-C contacts between *MECOM* and the strong promoter signal at *TERC* suggest common regulation of both cancer genes. Moreover, these data are consistent with variation affecting *TERC* as the genetic basis of the 3q26.2 association^43^. At 11q23, interactions with the region encoding AB231705 map to *C11orf53*, *C11orf92* and *C11orf93*, which have recently shown to be the functional basis of the 11q23 association^44^.

Overall, the chromatin contacts within the CRC risk interactome preferentially map to regulatory elements consistent with the tenet that many of the common CRC susceptibility loci influence transcriptional regulation networks. The significant improvement in effective resolution of cHi-C over conventional Hi-C allows us to identify interacting regions and refine association signals. In combination with additional high-resolution techniques, this should allow for delineation of specific interacting motifs. Our study therefore provides the basis for furthering our understanding of the mechanisms underscoring GWAS signals for complex diseases.

## Methods

### Definition of CRC risk loci

The strength of SNP associations at each of the CRC risk loci was defined from a previously published meta-analysis of five GWASs of Northern European ancestry totalling 5,626 CRC cases and 7,817 controls^18^.

### cHi-C experiments

The application of target sequence capture to the Hi-C protocol is outlined in Supplementary Fig. 26.

### Cell culture and formaldehyde crosslinking

Hi-C experiments were performed in three CRC cell lines, LS174T, LoVo and Colo205, grown in Eagle’s minimal essential medium (with 1% non-essential amino acids), Ham′s F12 and RPMI 1640, respectively, complemented with 2 mM glutamine and 10% fetal bovine serum. Cell lines were obtained from ‘Cancer Research UK Cell Services’, London, UK. Formaldehyde crosslinking of 10–30 million cells was performed by substituting standard culture media with fetal bovine serum-free media containing 2% formaldehyde for 5 min at room temperature. Crosslinking was quenched by addition of glycine to a final concentration of 125 mM. Adherent cells (LS174T and LoVo) were scraped off the culture flask after crosslinking. Cells were washed twice with cold PBS, snap-frozen in liquid nitrogen and stored at −80 °C before preparation of the Hi-C library.

### Hi-C library preparation

Hi-C library preparation, comprising cell permeabilization, chromatin fixation, HindIII digestion, biotin labelling, ligation and crosslink reversal was performed as described in van Berkum *et al.*^45^ with the following minor changes: (i) prior to biotin labelling, samples were incubated at 65 °C for 25 min with SDS, subsequently quenched by Triton-X and both reagents added at a final concentration of 1.3%. (ii) The final concentration of biotinylated dCTP during labelling was 0.025 mM. (iii) Following proteinase-K digestion of the post-ligation sample RNA was digested by addition of 40 μg ml^−1^ RNAse-A for 1 h at 37 °C. (iv) Following biotin pulldown, DNA was fragmented peaking at 500 bp. No fragment size selection was performed.

### Capture library design

A SureSelect Custom Target Enrichment Library covering the 14 CRC risk loci, represented by 18 tagSNPs, was designed using eArray software (Agilent, Santa Clara, CA, USA). Biotinylated RNA baits were generated to capture genomic sequence within LD blocks to which tagSNPs associations mapped (Supplementary Table 2). LD data were extracted using SNAP^46^ based on CEU HapMap^47^ phase 3, imposing parameters *r*^2^>0.2 and a minor allele frequency of >2%. LD measures for tagSNPs not included in HapMap were extracted from the thousand genomes (1000g) pilot data^48^. The region to be enriched encompassing the 8q24 risk locus was extended to 1.118 Mb centring on rs6983267. The total enrichment target of 4.683 Mb was submitted to Agilent eArray software, generating 43,380 120-mer RNA baits designed to tile the non-repetitive fraction of the test regions at 3 × coverage. Our design scale, tiling the total regions of interest after masking repeats, fitted the size ranges of commercially available Agilent SureSelect Custom Target Enrichment kits. Notably, designing baits for sequences >500–1,500 bp (depending on NGS fragment distribution) from a HindIII restriction site does not yield in improvement of the enrichment efficacy, which should be considered when facing limitations in bait numbers.

### Target enrichment

Target enrichment for the 15 test regions was performed based on the SureSelect protocol (Agilent) but incorporating the following modifications: (i) biotinylated Hi-C ditags bound to streptavidin beads were amplified pre-hybridization directly from beads using 7–10 PCR cycles in up to 96 parallel 50 μl reactions. Subsequently, PCR products were pooled, purified using Agencourt Ampure XP beads (Beckman Coulter, Brea, CA, USA) and concentrated using a speedvac concentrator to achieve the required input concentration for bait hybridization (500 ng). (ii) Enriched fragments were amplified post hybridization again directly from the streptavidin beads, using 10–12 cycles of PCR.

### Paired-end NGS

Three target-enriched Hi-C libraries, representing the CRC cell lines LS174T, LoVo and Colo205, were sequenced on multiple flow cell lanes on an Illumina HiSeq2000 (Illumina, San Diego, CA, USA) generating 50 or 100 bp paired-end reads.

### NGS read mapping

Sequencing data were processed through a custom pipeline formed of publicly available and in-house developed tools (Supplementary Fig. 26). Due to the nature of Hi-C ditags, single-end mapping was applied to the paired-end reads. Preliminary analyses of sequencing data comprising 100-bp reads showed that a length of 50 bases maximally generated uniquely aligned reads. Therefore, to obtain the required read depth for high-resolution analysis, libraries were re-sequenced on several flow cell lanes generating 50 base reads. Resulting FASTQ files from several sequencing patches were merged per cell line. Reads were mapped to the GRCh37/hg19 human genome assembly using Stampy v1.0.15 ref. 49 running the Burrows–Wheeler Aligner with standard single-end parameters. Re-establishment of the reads’ paired-end nature as well as paired-end-based removal of PCR duplicates was performed using Picard tools ( http://picard.sourceforge.net). Uniquely aligned read pairs were selected based on a mapping quality score threshold of MAPQ>30 (Stampy PHRED score). Details on read depth and filtering statistics of each cHi-C library are provided in Supplementary Table 7.

### Filtering for *bona fide* Hi-C contacts

Experimental background^50^, comprising circularized, non-digested and self-ligated fragments as well as fragments lacking HindIII restriction sites, was removed based on read orientation and distances to the nearest restriction sites. *Bona fide* Hi-C contacts are the fraction of the raw contact data set, which fulfil the criteria advocated (Supplementary Table 7).

### Analysis of Hi-C contacts

Adopting the notation of Lieberman-Aiden *et al.*^7^ the genome-wide ith row, jth column matrix entry is defined by the number of Hi-C contacts between locus i and locus j. A genome-wide coordinate system based on build GRCh37/hg19 was implemented. After removal of non-*bona fide* Hi-C contacts, each set of Hi-C ditags was allocated to the genome-wide enrichment contact matrix M_ij_. Because of target enrichment, data analysis necessitates ‘enriched versus enriched’ (E–E; that is, highly enriched for interaction counts), ‘enriched versus non-enriched’ (E–N; that is, enriched for interaction counts) and ‘non-enriched versus non-enriched’ (N–N; that is, not enriched for interaction counts) interactions to be processed separately (Supplementary Fig. 3). In our experiments bins were defined as blocks of 9 or 3 kb. Bins populated by entries of the contact matrix were filtered according to pre-loaded mapability and restriction fragment tracks. Only valid bins of E–E and E–N, containing HindIII sites and having an average mapability >0.5, were considered for further analysis. We used a moving window (that is, 10 Mb, size restricted by computational power) to bin the contacts and to generate the genome-wide interaction matrix split into heatmaps (Supplementary Fig. 1).

### Correction of bias in cHi-C

Local structures of the genome such as location of restriction sites in respect to bins (fragment length bias), GC content and mapability can influence Hi-C contact frequency resulting in bias^37^,^50^. Furthermore, binning at high resolutions close to the average restriction fragment length may result in bias augmentation introduced by the unequal distribution of restriction sites over bins. To adjust for such biases, binned Hi-C contact matrices were normalized based on principles previously articulated^24^,^35^,^37^,^51^, but adapted to the target-enriched setting. To normalize the data, we computed for each bin *i* its weight *w*_*i*_ by counting the number of reads mapping to bin *i* at *trans-*loci across the genome. The quantity *w*_*i*_ was computed for each column of the genome-wide contact matrix. Similarly, the total number *N*_*i*_ of populated contact matrix entries for each bin was computed. The total number of contacts and populated bins, *W*_*i*_ and *A*_*i*_, respectively, were determined. Finally, the normalized weights *ŵ*_*i*_=*w*_*i*_*·A*_*i*_/(*N*_*i*_*·W*_*i*_) were computed and used to normalize matrix entries *M*_*ij*_ (Supplementary Equation 1). The resulting recursion relation (Supplementary Equation 1) defining our bias normalization procedure updated these weights, under iteration, until convergence was achieved. To ensure convergence of all *ŵ*_*i*_ to unity within the specified precision, bins with initial weights *w*_*i*_ more than three s.d. below the mean weight were discarded prior to starting the recursion relation. For computational reasons, we performed bias normalization adopting a moving window of 10 Mb (Supplementary Fig. 1).

### Distance normalization of cHi-C data

After bias normalization, each contact between a pair of inter-chromosomal loci was distance normalized according to the expected contact frequency. Consistency between contact frequency profiles computed by applying a weighted average-smoothing procedure on different chromosomes and/or target-enriched regions resulted in a template contact frequency profile (Supplementary Fig. 2). The scaling behaviour, previously reported^7^, and characterized by a power law with an estimated exponent of −1.08 in a wide range of distances (0.5 Mb≤*d*≤7 Mb) was confirmed in our study, where we measured an exponent of −0.97. Over low distance, 9 kb<*d*<0.5 Mb, a different exponent value, −0.52, was observed (Supplementary Fig. 2). The contact frequency profile was used to distance-normalize the interaction frequency of each target bin. To avoid over-correction in *cis* outside the test regions, the degree of coverage at sites of interactor bins was used to adjust contact frequency counts. Specifically, if coverage was low, distance normalization was less penalizing.

### Significance of cHi-C interactions

Identifying biologically important chromatin interactions above experimental background requires Hi-C contacts significantly stronger than expected by chance. Aiming to identify the fraction of functional, TF-mediated contacts, a parametric statistical model was fitted to the distribution of the Hi-C data, assigning *P* values to contact frequencies. Test, *cis* and *trans-*regions were analysed separately fitting a zero-inflated Weibull distribution to the bias and distance-normalized contact frequencies. For the test region, all Hi-C contacts were pooled, whereas for *cis* and *trans* a distribution was fitted separately for each interval, using standard maximum likelihood techniques to estimate distributional parameters. To avoid the significant Hi-C contacts impacting on parameter estimation, the Weibull part of the zero-inflated Weibull distribution was truncated to the lowest 95 percentiles during parameter estimation. Some of the fitted distributions are shown in Supplementary Fig. 25. A *P* value for a given cHi-C contact was calculated as the probability of observing an equally strong or stronger contact under the fitted zero-inflated Weibull distribution. To adjust *P* values for multiple testing, we computed FDR *q*-values^52^. A cHi-C contact was deemed significant if it is corresponding *q*<0.05. To mitigate against cell-line-specific interactions, here we considered only contacts achieving a *q*<0.05 in all three CRC cell lines LS174T, LoVo and Colo205 (Supplementary Tables 3 and 4). The *P* value for the overlap of chromatin interactions significant in the three cell lines was obtained by computing the probability of observing an overlap of *k* or more elements between three independent samples, each sample *s*_*i*_ consisting of *n*_*i*_ elements, sampled from a set *S*_*i*_ of *N*_*i*_ elements, *i*=1, 2, 3, with *k*≤min_i_{*n*_*i*_} and *S*_*1*_⊆*S*_*2*_⊆*S*_*3*_. Assuming independence, not accounting for potential biases remaining after the applied normalization for experimental and distance biases, we observed a non-random occurrence of *k*=147 overlaps between the three data sets, *n*(LS174T, LoVo and Colo205)=216379, 177893 and 217396, respectively, and *N*(LS174T, LoVo and Colo205)=97376960, 95608750 and 92912739, respectively. To examine our ability to identify CRC-specific interactions at 8q24.21, we made use of in-house cHi-C data generated for SUM44 and GM12878 cell lines, as well as publicly accessible Hi-C data on IMR90 (ref. 53).

### Calculation of enrichment factor

The enrichment factor is defined as the ratio of on-target reads in a cHi-C test library (LS174T, LoVo and Colo205, respectively) to that in a conventional Hi-C reference library. Here we made use of a publicly available library in GM06990 cells^7^. PCR duplicates do not increase linearly with increasing library size; hence it is essential to size-match (in terms of numbers of raw NGS reads) test and reference libraries. NGS fragments randomly populate a flow cell, and random subsets of reads were obtained by randomly selecting a sub-area of the flow cell. The same mapping and filtering protocols (described above) that were applied to the full libraries (Supplementary Table 7) were then applied to the selected subsets (Supplementary Table 1). In addition, we estimated enrichment by calculating the average read count per 9 kb bin in the off-target reads compared with the average read count per bin in the on-target reads (Supplementary Table 1).

### Validation of significant cHi-C contacts

To technically validate cHi-C results, we applied 4C-seq^38^ to examine close-*cis-* (<5 Mb) interactions at four of the 14 loci (Supplementary Table 8). The 4C-seq experimental procedures are described in detail in the Supplementary Methods. In addition, seven far-*cis-* (>5 Mb) and seven *trans-*interactions (Supplementary Table 3) were validated using interphase FISH as an orthogonal methodology^4^. Details on the FISH experiment and determination of co-localization frequencies through automated image analysis are described in the Supplementary Methods.

### Refining interaction domains at high effective resolution

A directionality index (DIX) for each bin was determined by quantifying the bias of a bin to interact upstream/downstream. The null distribution of the DIX statistic *D*, under the assumption of no bias, is related to a *χ*^2^-distributed with 1 d.f.^25^, where *D*=|*A−B*|·(B−A)/(A+B) and *A*/*B* is the number of upstream/downstream contacts within 495 kb from a given 9 kb bin, respectively. Hi-C domain caller software^25^ was used to obtain topological domains from the DIX using a hidden Markov model.

### Evaluating chromatin interactions for association overlap

Statistically significant chromatin interactions at 9 k resolution (Supplementary Data 1) were evaluated for overlap with high association signals from typed and imputed SNPs^18^ ( http://tinyurl.com/whiffinetal2013). The *P* value assigned to the observation of significant chromatin interactions being preferentially built by regions of strong CRC risk association was calculated as follows: the 15 test regions (Supplementary Table 2) are covered by a total of 366 test bins passing the mapability filter at 9 kb resolution. Out of those, 88 bins (24%) overlap low association *P* values (one order of magnitude within the region’s lowest *P* value), whereas out of the 61 significant test bins, 23 bins (38%) overlap low association *P* values (8.54 × 10^−3^, Fisher’s exact test; Supplementary Table 4).

### Annotating the epigenetic pattern at cHi-C contacts

We used ChromHMM^54^ to infer and characterize chromatin states by integrating information on histone modifications to identify combinatorial and spatial patterns of epigenetic marks. Aligned NGS reads (BAM format) from ChIP-Seq and DNAse-Seq experiments on the CRC cell line HCT116 (Supplementary Table 9) were downloaded from ENCODE^55^,^56^. Data consisting of replicates performed within and between different laboratories belonging to the ENCODE project were combined using WIGGLER (a.k.a. align2rawsignal)^57^. Read-shift parameters for ChIP-Seq data were calculated using PHANTOMPEAKQUALTOOLS^58^. Using the ChromHMM software, genome-wide signal tracks were binarized (including input controls for ChIP-Seq data), and a set of learned models, using 43 random initializations with 32 different states, were generated on a representative chromosome (chr8). The parameters of the highest scoring model were retained and model states were pruned from 32 to 2 states. A 27-state ChromHMM model was shown to be stable using Emission Parameter Correlation Comparison and was subsequently used for segmenting the genome at 200 bp resolution (Supplementary Fig. 27). We also trained ChromHMM with 64 different states, using 45 random initializations, but found no advantage of segmenting the data with this increased number of states, models with 32 and 64 different states being consistent with each other.

### Evaluation for overlap with regulatory chromatin segments

Genome-wide chromatin interactions at 9 kb resolution (Supplementary Data 1) were evaluated for overlap with regulatory elements using two approaches. First, focusing on the CRC cell line HCT116, we determined whether regulatory elements (enhancers and promoters) are present at higher frequency within chromatin-looping interactors than expected at random size-matched sites of the genome. A Monte Carlo procedure was used, taking 10^7^ random samples of 61 (number of significantly interacting test bins) 9 kb bins along the genome. For each of these *N* samples and for each class c of regulatory element (c=promoter or enhancer), the proportion of bins overlapping with regulatory elements of class c was computed. A *P* value (for the null hypothesis of no increase in overlap proportion with class c) resulted from the fraction *m*_c_*/N* where *m*_c_=the number of samples with overlap proportions larger than or equal to the observed overlap proportion. If *m*_c_=0, a *P* value <1/*N* is reported. Second, tissue specificity of overlaps was examined comparing the CRC cell line HCT116 with nine other cell types (Supplementary Table 6). Briefly, ChromHMM-based chromatin segmentation data for Gm12878, H1hesc, Hepg2, Hmec, Hsmm, Huvec, K562, Nhek and Nhlf were retrieved from the ENCODE database^52^,^53^ (Supplementary Table 6). Downloaded chromatin segmentations were regrouped to a simplified four-state scheme (Supplementary Table 10) to allow for direct comparison (Fisher’s exact test, Supplementary Table 6).

### Depicting evolutionary conservation profiles

To evaluate potential functional variants within candidate causative elements (Supplementary Table 5), we used phastCons^59^ (derived from sequence comparison of 46 vertebrates ) and genomic evolutionary rate profiling (GERP)^60^,^61^ scores as measures for evolutionary conservation. The phastCons score reflects the probability that a given nucleotide is conserved; the score ranges from 0 to 1, where 1 is most conserved. The GERP score (range −12.36 to 6.18) reflects position-specific constraint, positive scores scaling with the level of constraint such that higher scores indicate a greater level of evolutionary conservation.

### Evaluating chromatin interactions for TF binding

To test whether specific TF binding underlies significant chromatin interactions, we integrated the CRC chromatin interactome data with TFBS profiles derived from LoVo cells. Peak files generated from ChIP-Seq experiments for 433 TFs were screened for TFBSs shared between test bins and interactor bins of each Hi-C contact. Eighty-nine of the 147 significant genome-wide Hi-C interactions at 9 k resolution shared at least one of the assayed TFs. Hundred out of the total 433 TFs were found to be part of a shared cluster (Supplementary Table 11). To test whether Hi-C interactions are more likely to comprise shared TF binding compared with random 9-kb control bins of comparable interactivity, control interaction pairs were modelled by permutation (total *n*=147; Supplementary Data 1), excluding pairs from the same test regions. The Hi-C interaction pairs were tested against the permuted control set using a Mann–Whitney *U*-test (Supplementary Fig. 23).

### Statistical analyses and visualization

All statistics were performed using the R suite^62^. Multi-track data on cHi-C interactions and associated genetic and epigenetic features were visualized using visPIG^63^.

## Author contributions

R.J., P.F., O.F. and R.S.H. contributed with conception and experimental design. R.J., R.K. and H.E.S. helped with acquisition of data. R.J., G.M., M.H., R.K., H.E.S., A.H. and N.W. carried out analysis and interpretation of data. M.J.C., L.B., N.D., T.N., S.S., M.E. and Y.Y. provided administrative, technical or material support. R.J., G.M,. M.H., A.H., J.T., P.F., O.F. and R.S.H. wrote the manuscript. R.S.H. supervised the study.

## Additional information

**Accession codes.** Sequencing data have been deposited in the European Genome-phenome Archive (EGA), which is hosted by the European Bioinformatics Institute, under the accession code EGAS00001001085.

**How to cite this article:** Jäger, R. *et al.* Capture Hi-C identifies the chromatin interactome of colorectal cancer risk loci. *Nat. Commun.* 6:6178 doi: 10.1038/ncomms7178 (2015).

## Supplementary Material

Supplementary Information Supplementary Figures 1-27, Supplementary Tables 1-11, Supplementary Method and Supplementary References.

Supplementary Dataset 1 Long-range interactions at 9kb resolution for LS174T, LoVo and Colo205 filtered at a 5% FDR (q<0.05) in all of the three cell lines. Genomic positions are based on GRCh37/hg19 human genome assembly. Start positions of 9kb bins are provided. P-values are based upon a parametric test using a zero-inflated Weibull distribution.

Supplementary Dataset 2 Long-range interactions in close-cis at 3kb resolution for LS174T, LoVo and Colo205 filtered at a 5% FDR (q<0.05) in each cell line. Genomic positions are based on GRCh37/hg19 human genome assembly. Start positions of 3kb bins are provided. P-values are based upon a parametric test using a zero-inflated Weibull distribution.
