# Supplementary material for: Capture Hi-C identifies the chromatin interactome of colorectal cancer risk loci
Source: Nat Commun. 2015 Feb 19;6:6178. doi: 10.1038/ncomms7178 (PMC4346635; doi:10.1038/ncomms7178)
Supplement: Supplementary Information — Supplementary Figures 1-27, Supplementary Tables 1-11, Supplementary Method and Supplementary References. [file ncomms7178-s1.pdf]

SUPPLEMENTARY INFORMATION

Supplementary Figures

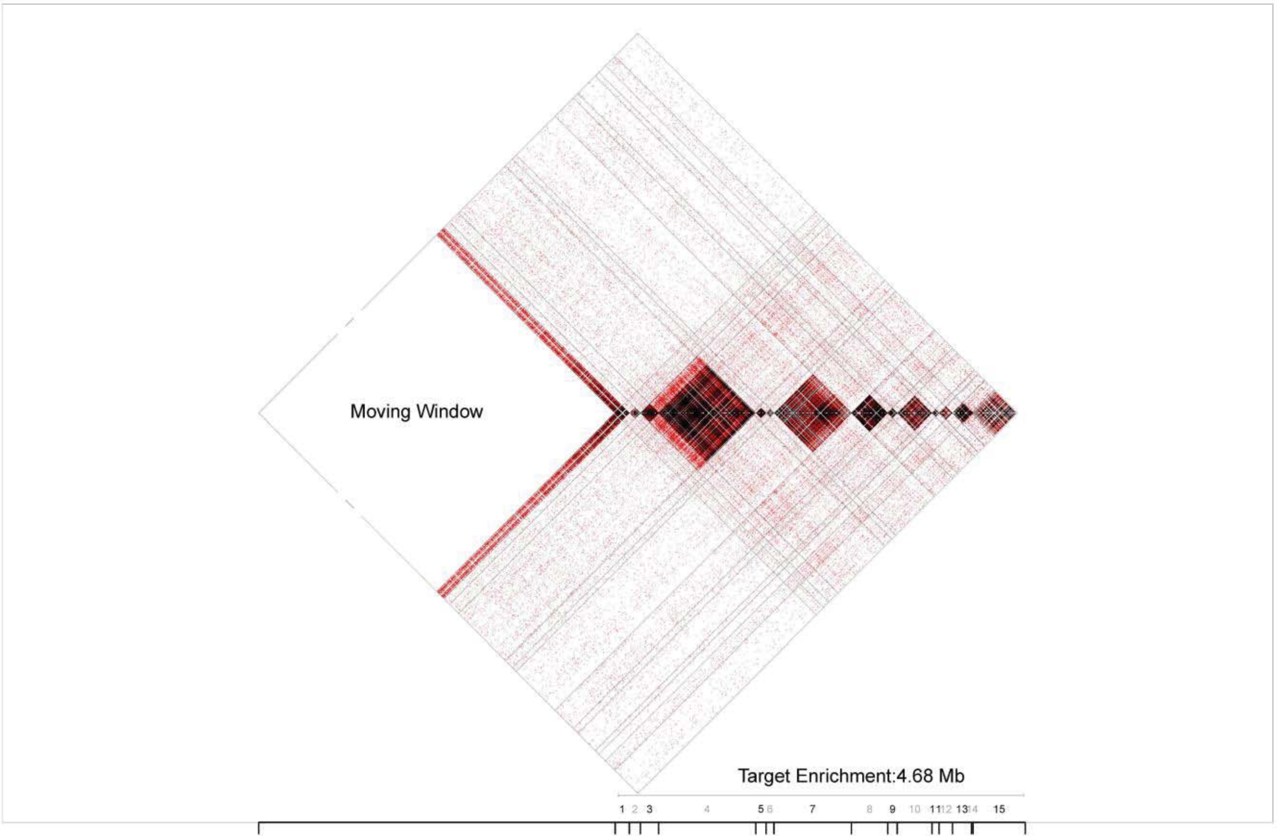

Supplementary Figure 1: Heatmap of genome-wide interaction matrix. Here we have applied a moving window to split the genome-wide interaction matrix into heatmaps. Shown is a representative heatmap based on a window (N-N) upstream of test region ENR_01 on chromosome 1. Only E-N (dark red stripe in *cis* and light red area in *trans*) and E-E (dark red squares) bins are stored.

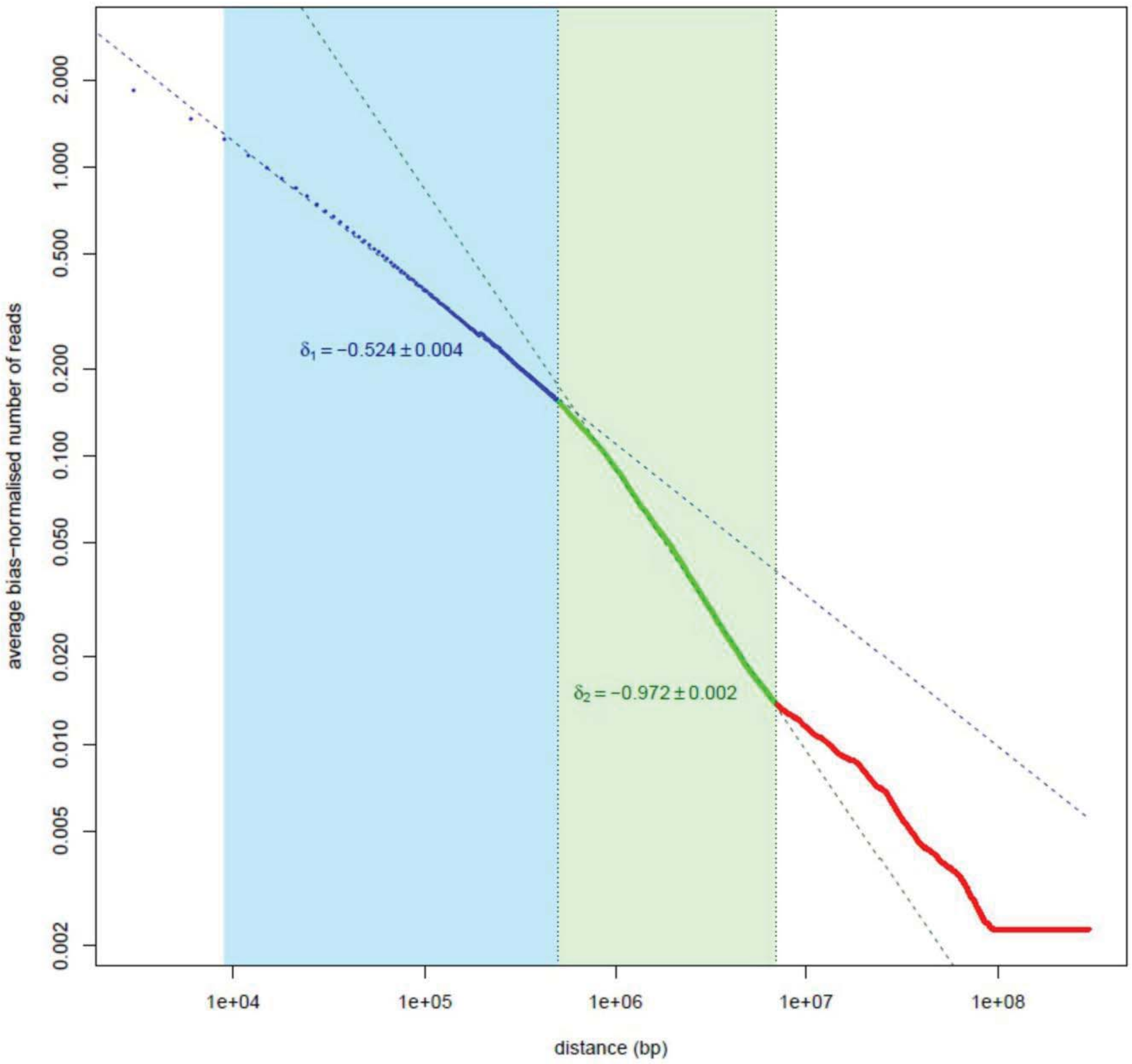

Supplementary Figure 2: Intensity profile of contact frequencies over distance. Shown is the crossover from a power law with exponent ≈ -1 for distances of 1Mb ≤ d ≤ 10Mb to a power law with exponent ≈ -0.5, for distances 9kb ≤ d ≤ 1Mb. The profile was obtained by a weighted average smoothing procedure.

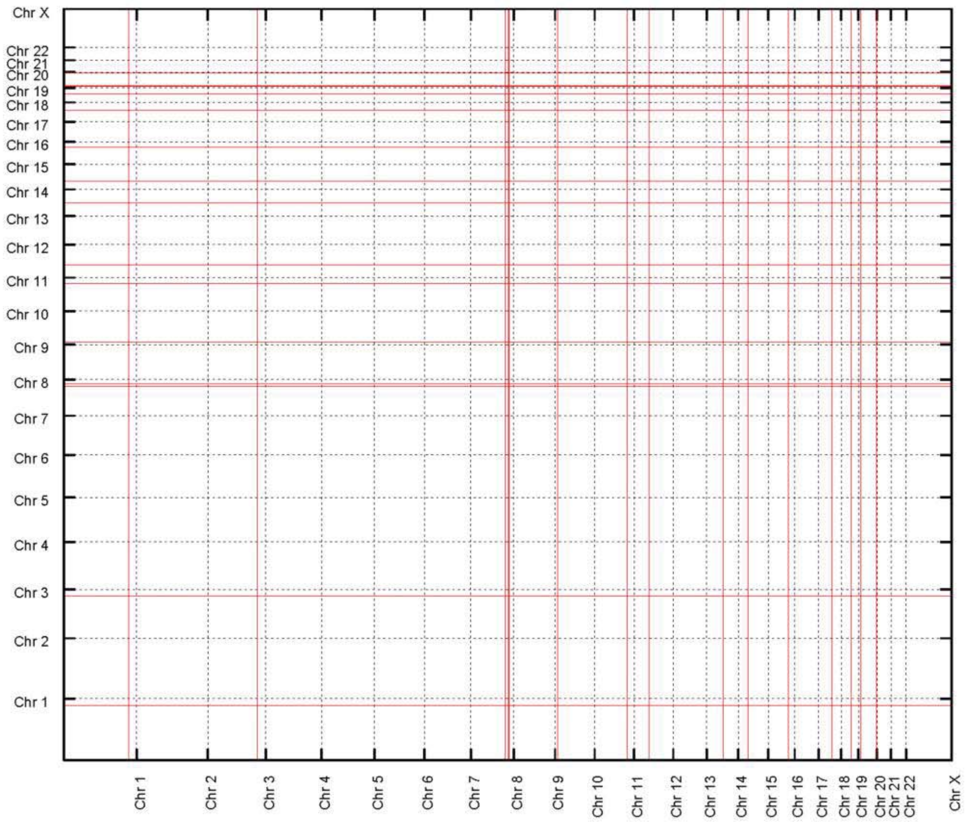

Supplementary Figure 3: Genome-wide contact matrix of enriched target regions. Captured test regions are shown as a red grid in context of the whole genome. Red lines represent "enriched vs. non-enriched" (E-N), their crossing points are "enriched vs. enriched" (E-E), whereas white space is "non-enriched vs. non-enriched" (N-N), the latter being excluded from analysis. Given a total target enrichment region $G' \approx 4.68$Mb, a genome-wide region of area $G' \cdot g \approx 1.41 \times 10^{16}$ bp² was retained, where g is the size of the genome (~3 Gb). Discarding off-target interactions (N-N) reduces the effective area spanned by genome-wide contacts to ~0.16% of the total.

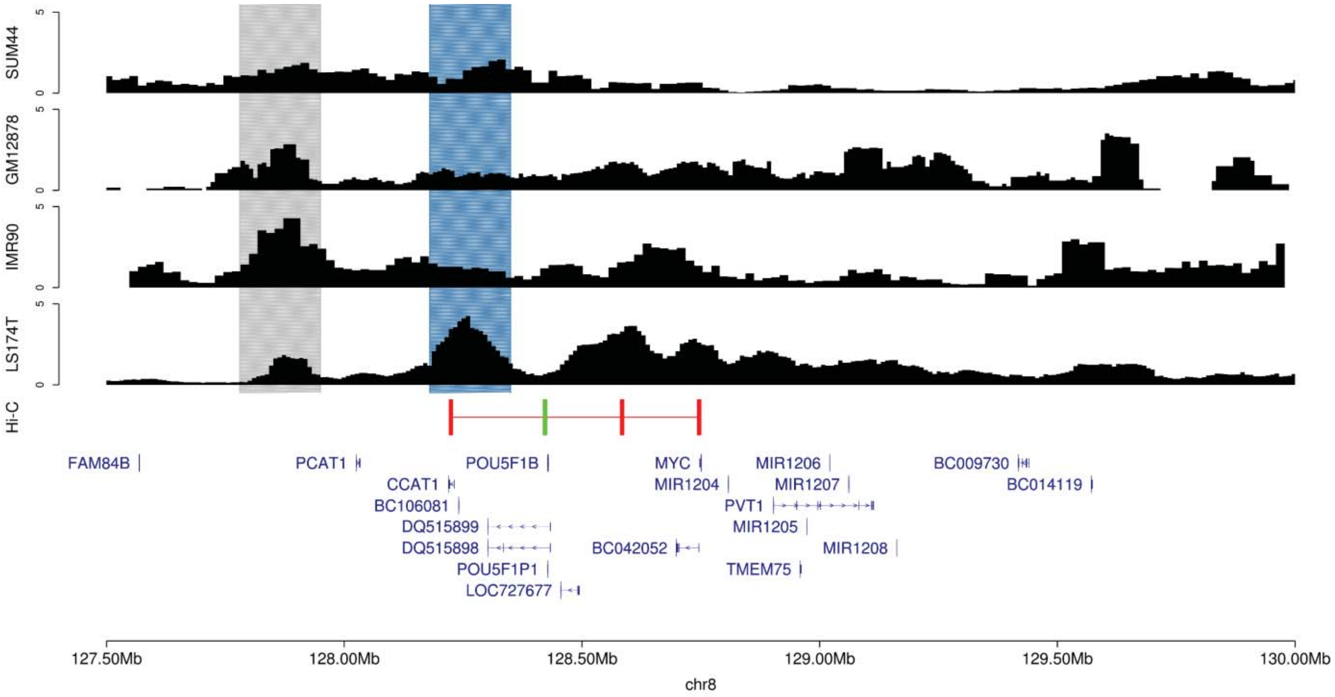

Supplementary Figure 4: 8q24.21 chromatin interaction profiles for LS174T, SUM44, GM12878, IMR90 cell lines for the viewpoint centromeric to *POU5F1B*. Shown in a shaded grey box is an interaction peak which appears to be generic, whereas the peak indicated by the light blue shaded box appears to be specific to CRC.

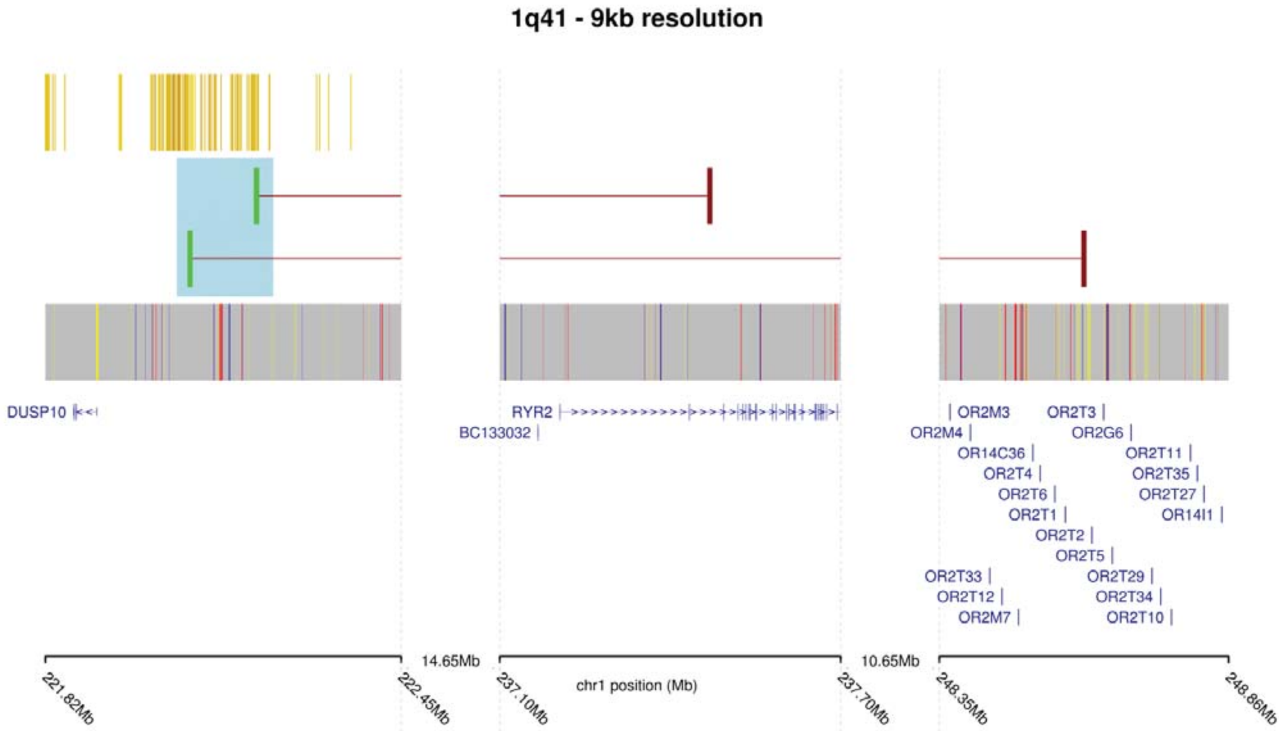

Supplementary Figure 5: Genome-wide annotation of significant chromatin interactions at 1q41 at 9kb resolution. Please refer to the legend from Figure 2.

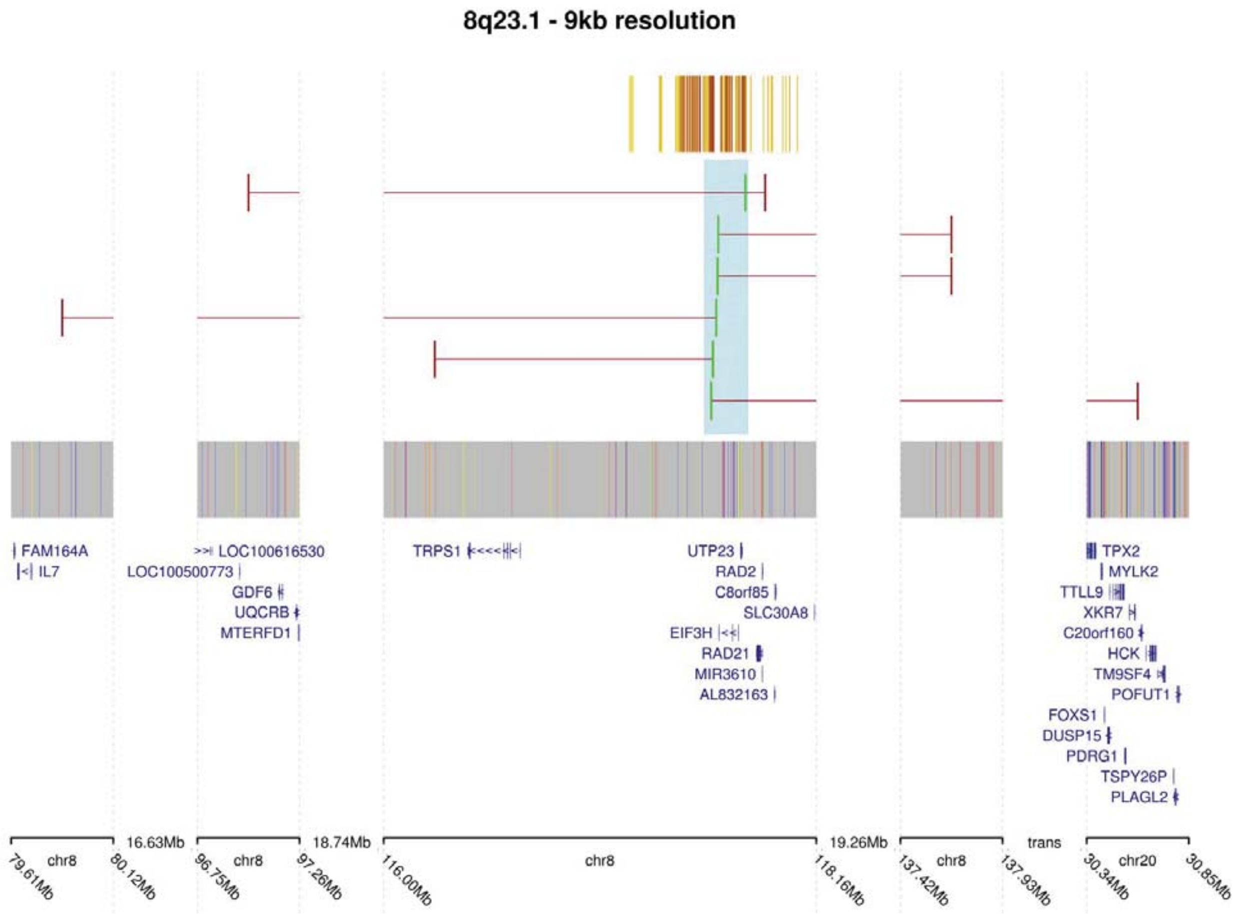

Supplementary Figure 6: Genome-wide annotation of significant chromatin interactions at 8q23.1 at 9kb resolution. Please refer to the legend from Figure 2.

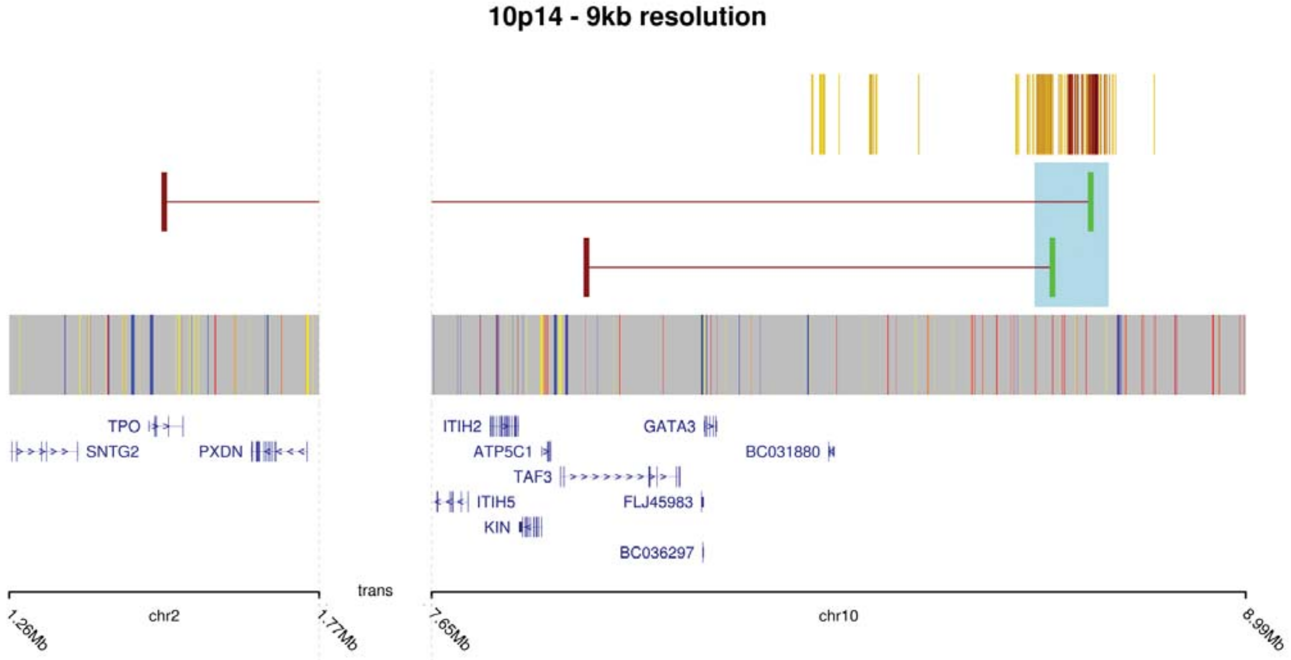

Supplementary Figure 7: Genome-wide annotation of significant chromatin interactions at 10p14 at 9kb resolution. Please refer to the legend from Figure 2.

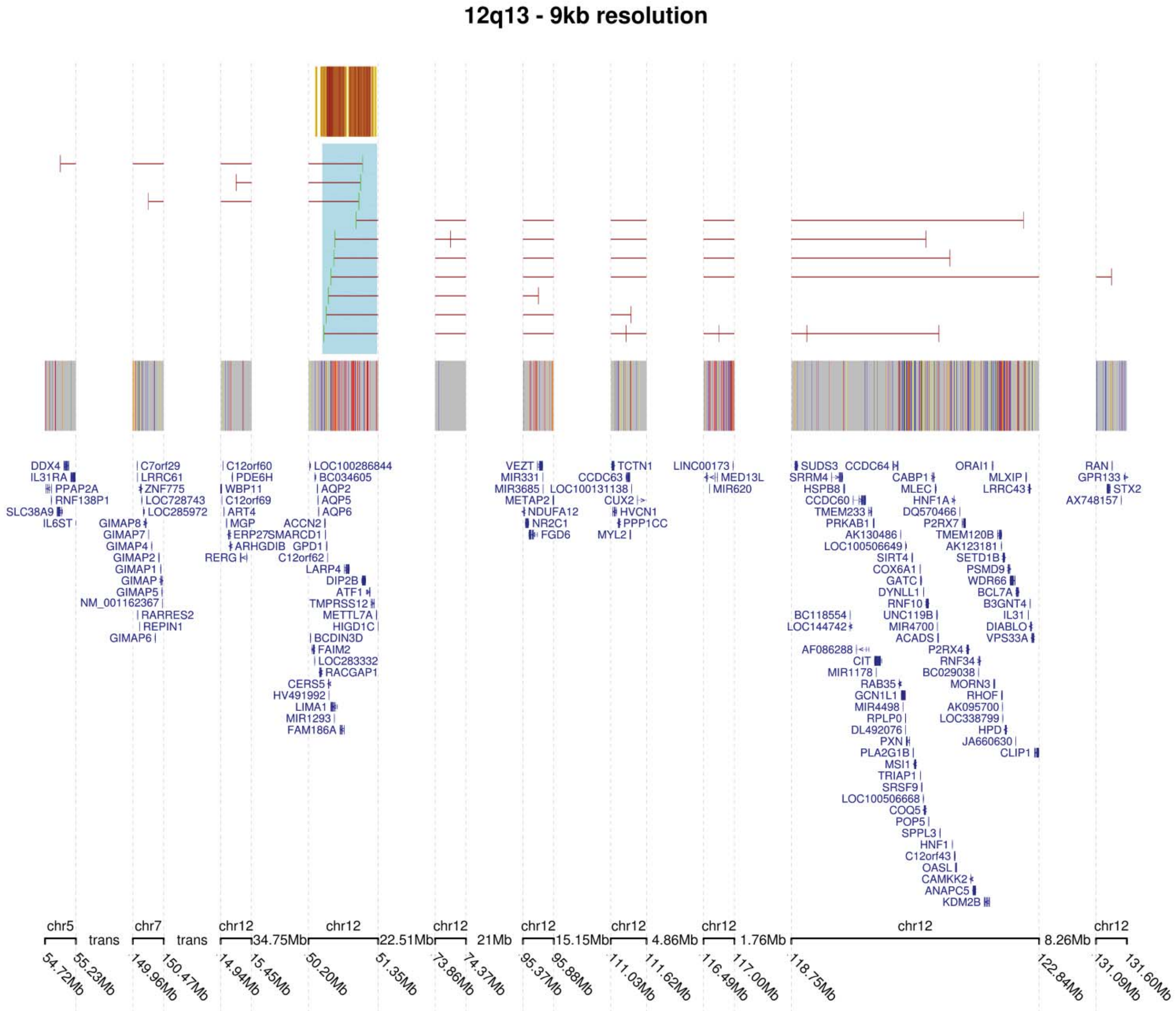

Supplementary Figure 8: Genome-wide annotation of significant chromatin interactions at 12q13 at 9kb resolution. Please refer to the legend from Figure 2.

14q22.2 - 9kb resolution

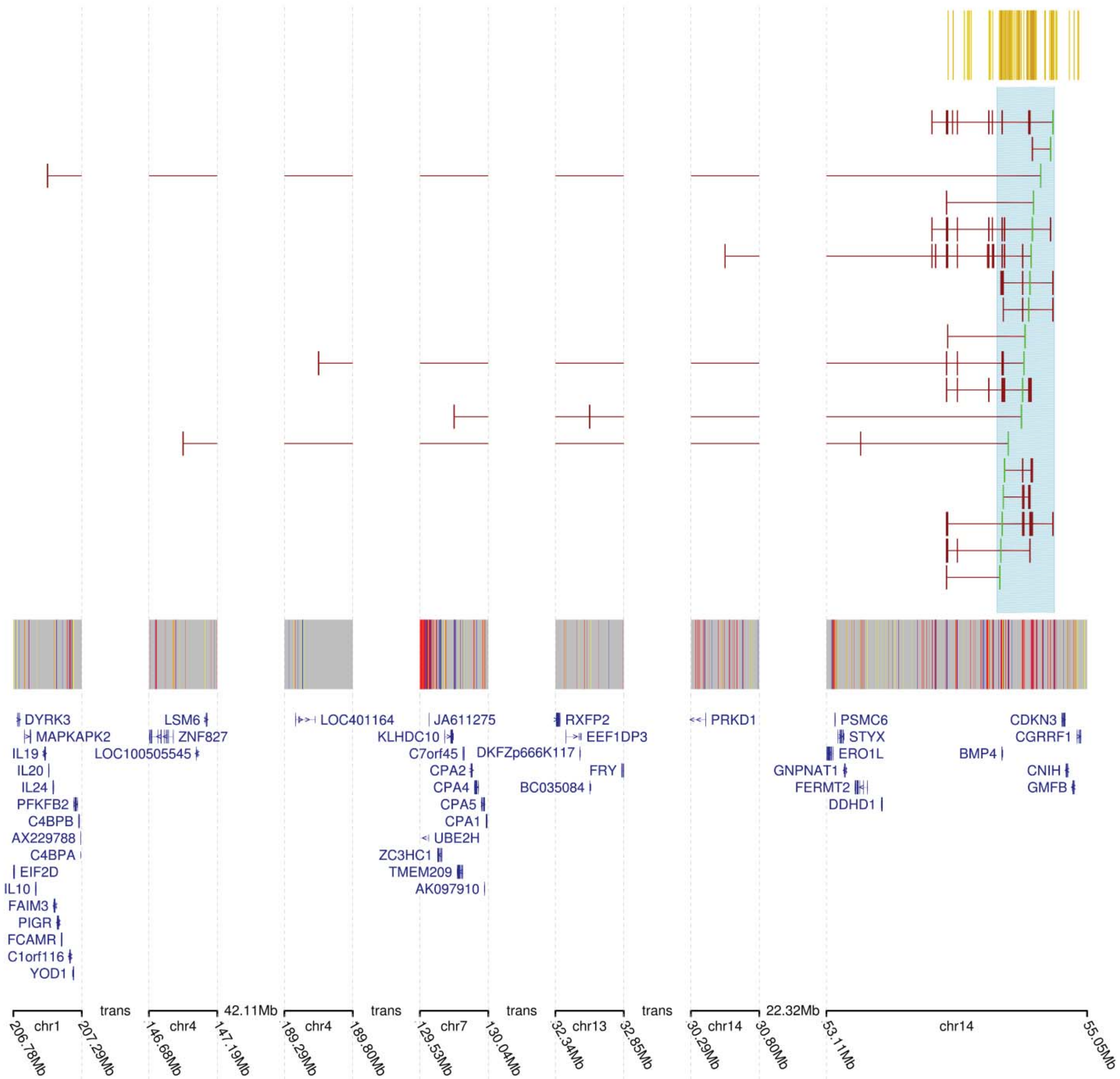

Supplementary Figure 9: Genome-wide annotation of significant chromatin interactions at 14q22.2 at 9kb resolution. Please refer to the legend from Figure 2.

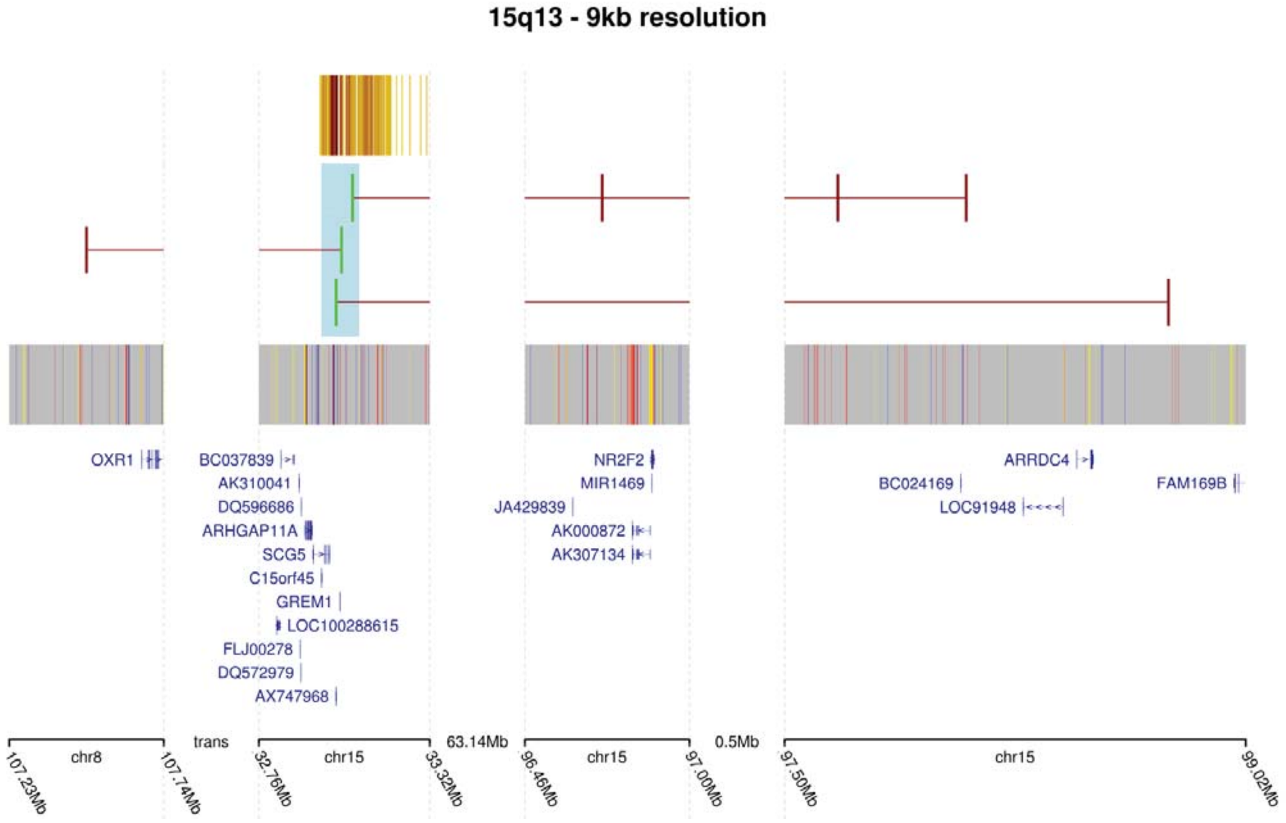

Supplementary Figure 10: Genome-wide annotation of significant chromatin interactions at 15q13 at 9kb resolution. Please refer to the legend from Figure 2.

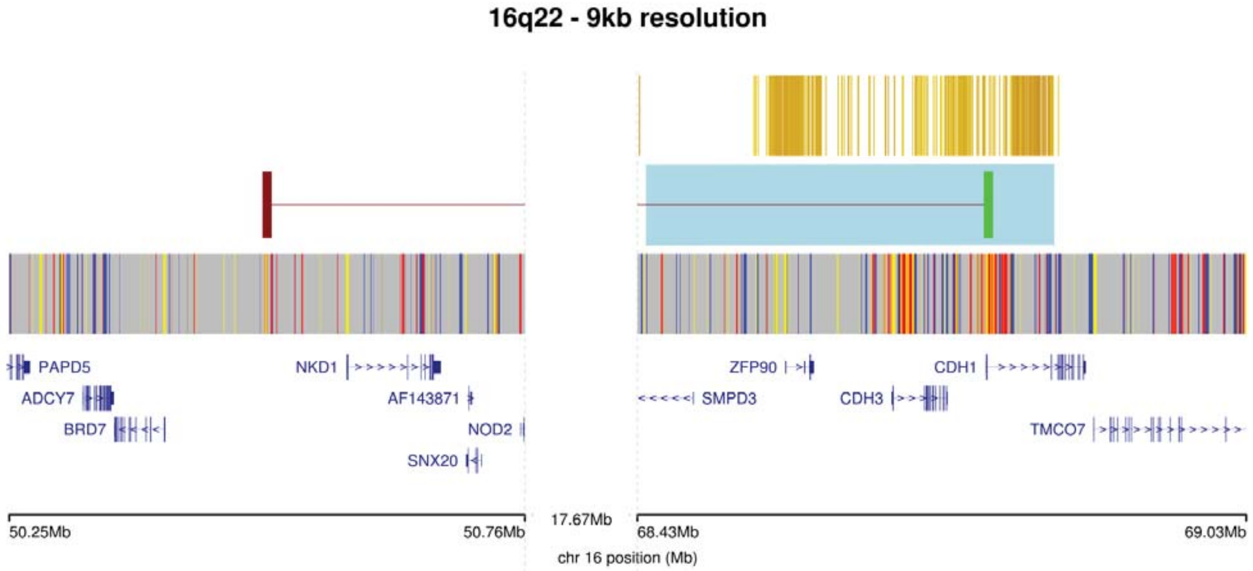

Supplementary Figure 11: Genome-wide annotation of significant chromatin interactions at 16q22 at 9kb resolution. Please refer to the legend from Figure 2.

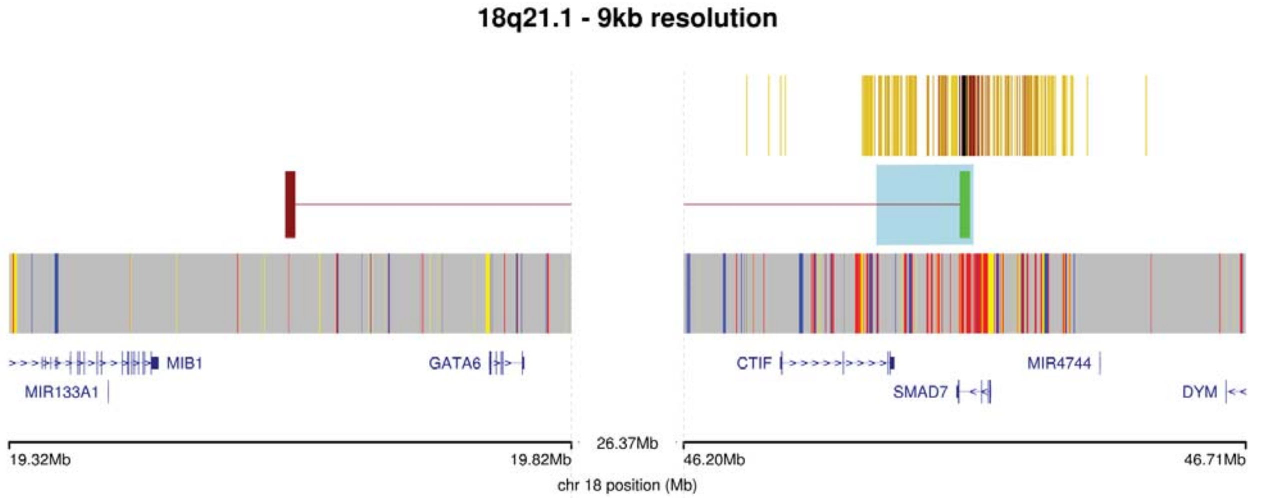

Supplementary Figure 12: Genome-wide annotation of significant chromatin interactions at 18q21.1 at 9kb resolution. Please refer to the legend from Figure 2.

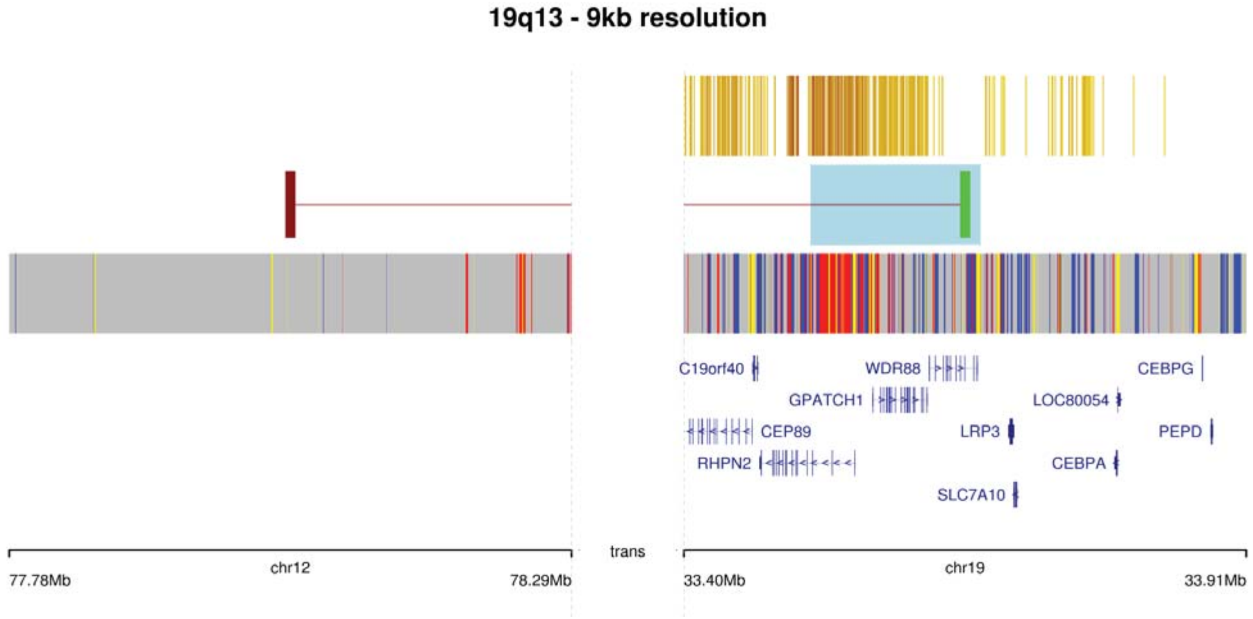

Supplementary Figure 13: Genome-wide annotation of significant chromatin interactions at 19q13 at 9kb resolution. Please refer to the legend from Figure 2.

20p12.3 - 9kb resolution

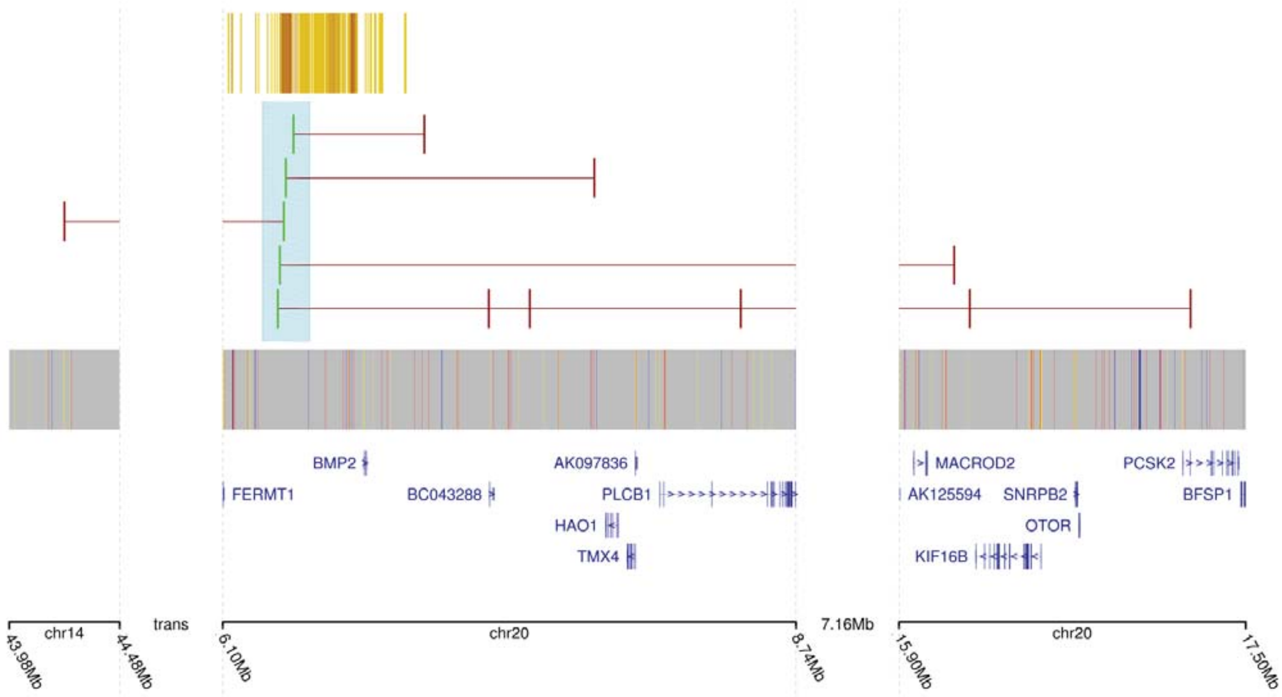

Supplementary Figure 14: Genome-wide annotation of significant chromatin interactions at 20p12.3 at 9kb resolution. Please refer to the legend from Figure 2.

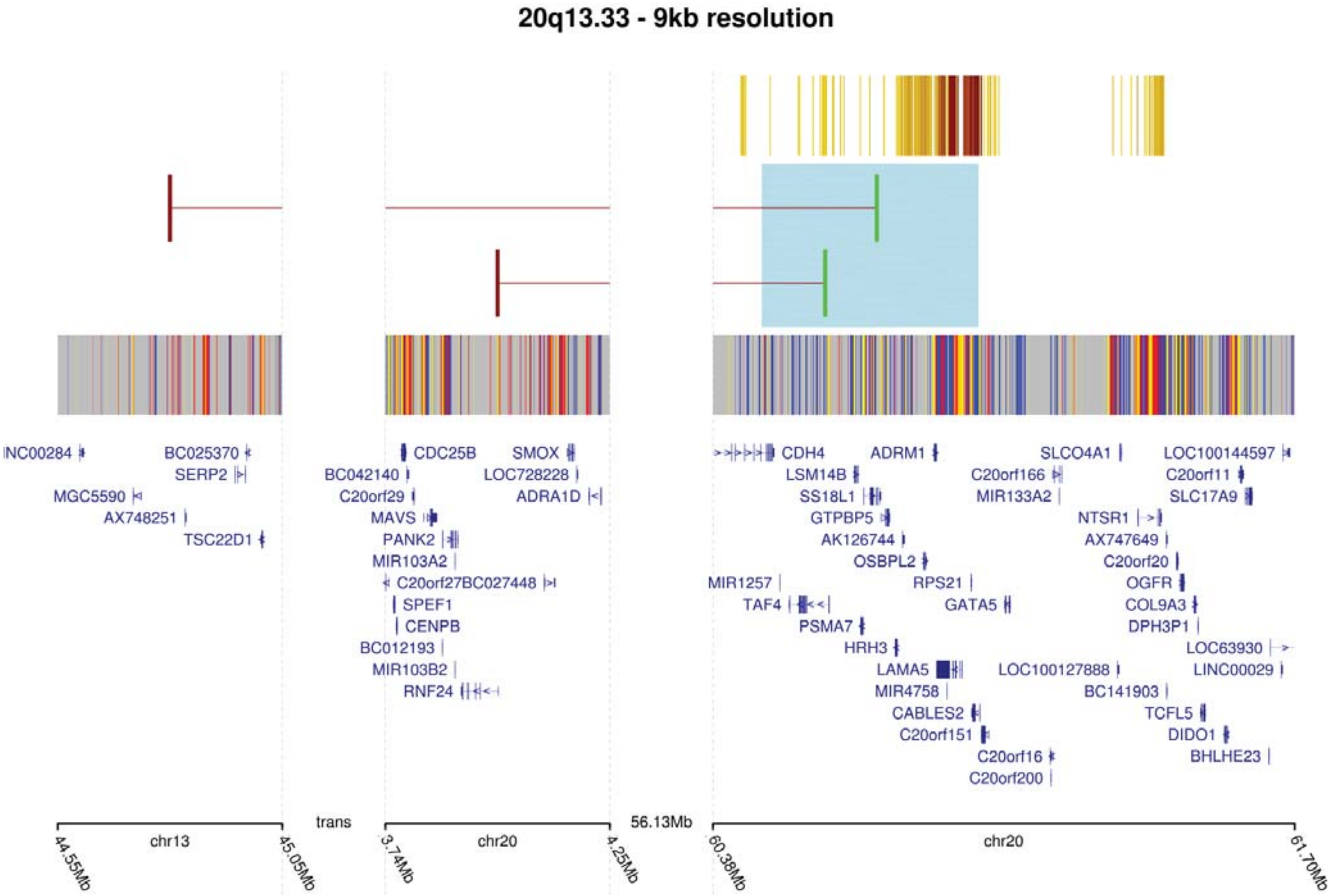

Supplementary Figure 15: Genome-wide annotation of significant chromatin interactions at 20q13.33 at 9kb resolution. Please refer to the legend from Figure 2.

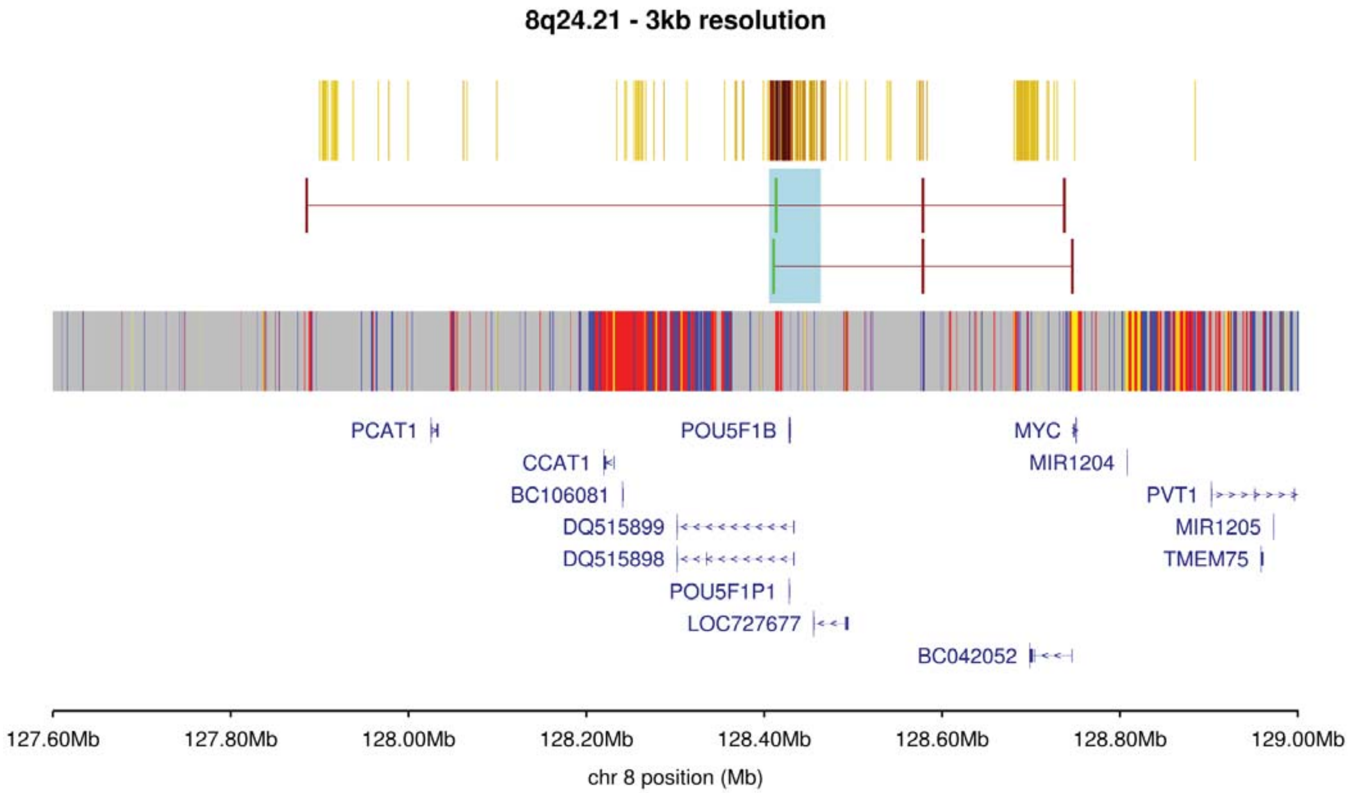

Supplementary Figure 16: Close-*cis* (+/- 5Mb) annotation of significant chromatin interactions at 8q24.21 at 3kb resolution. Please refer to the legend from Figure 2.

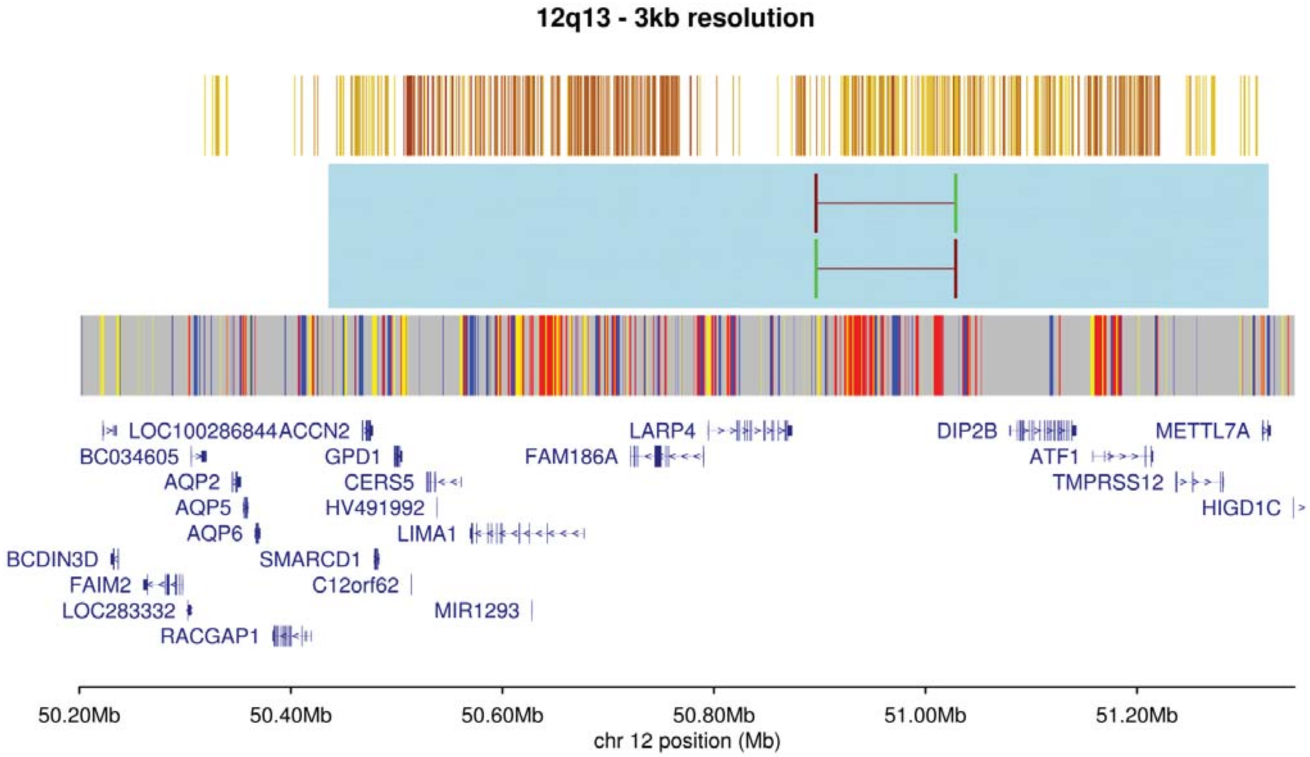

Supplementary Figure 17: Close-*cis* (+/- 5Mb) annotation of significant chromatin interactions at 12q13 at 3kb resolution. Please refer to the legend from Figure 2.

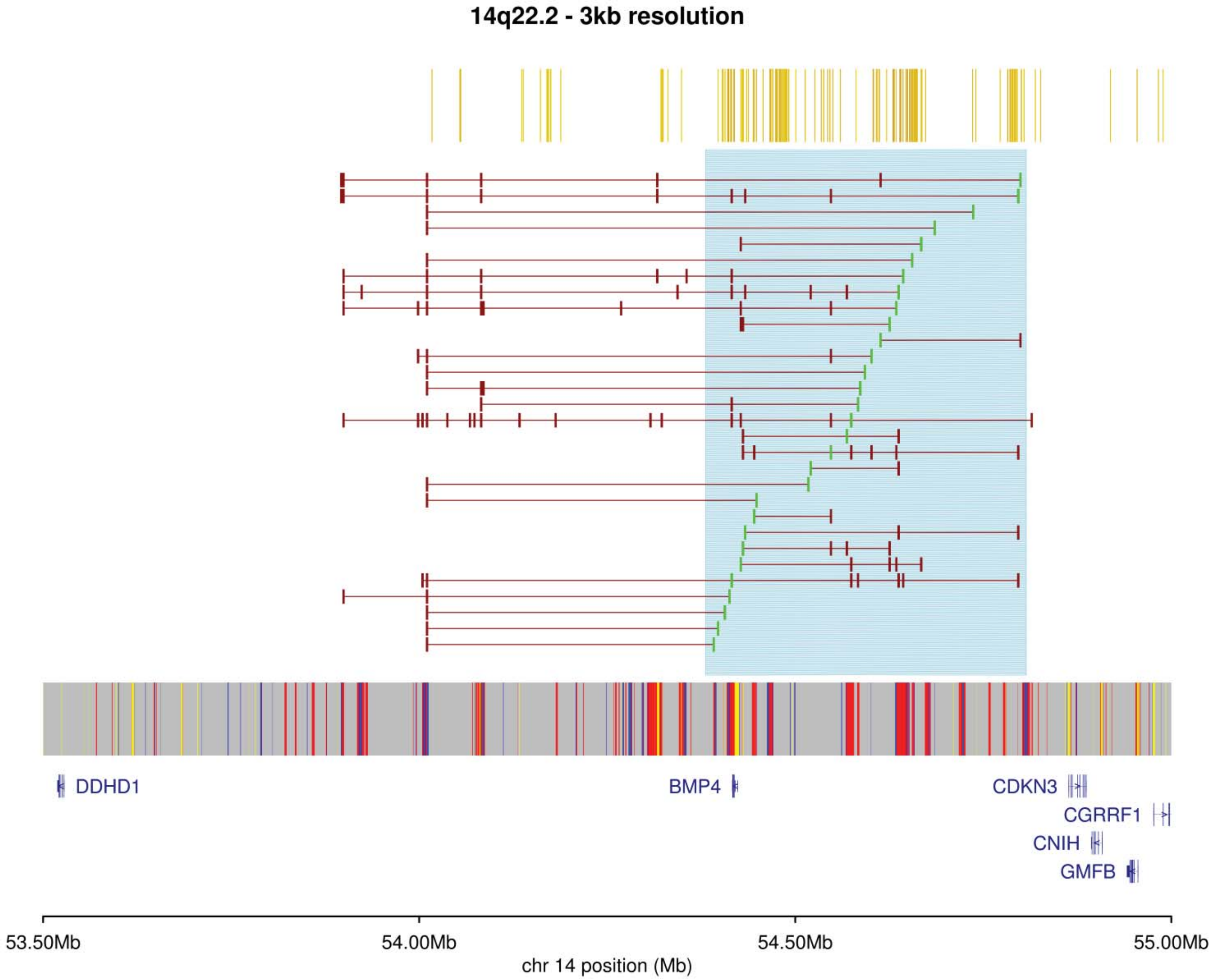

Supplementary Figure 18: Close-*cis* (+/- 5Mb) annotation of significant chromatin interactions at 14q22.2 at 3kb resolution. Please refer to the legend from Figure 2.

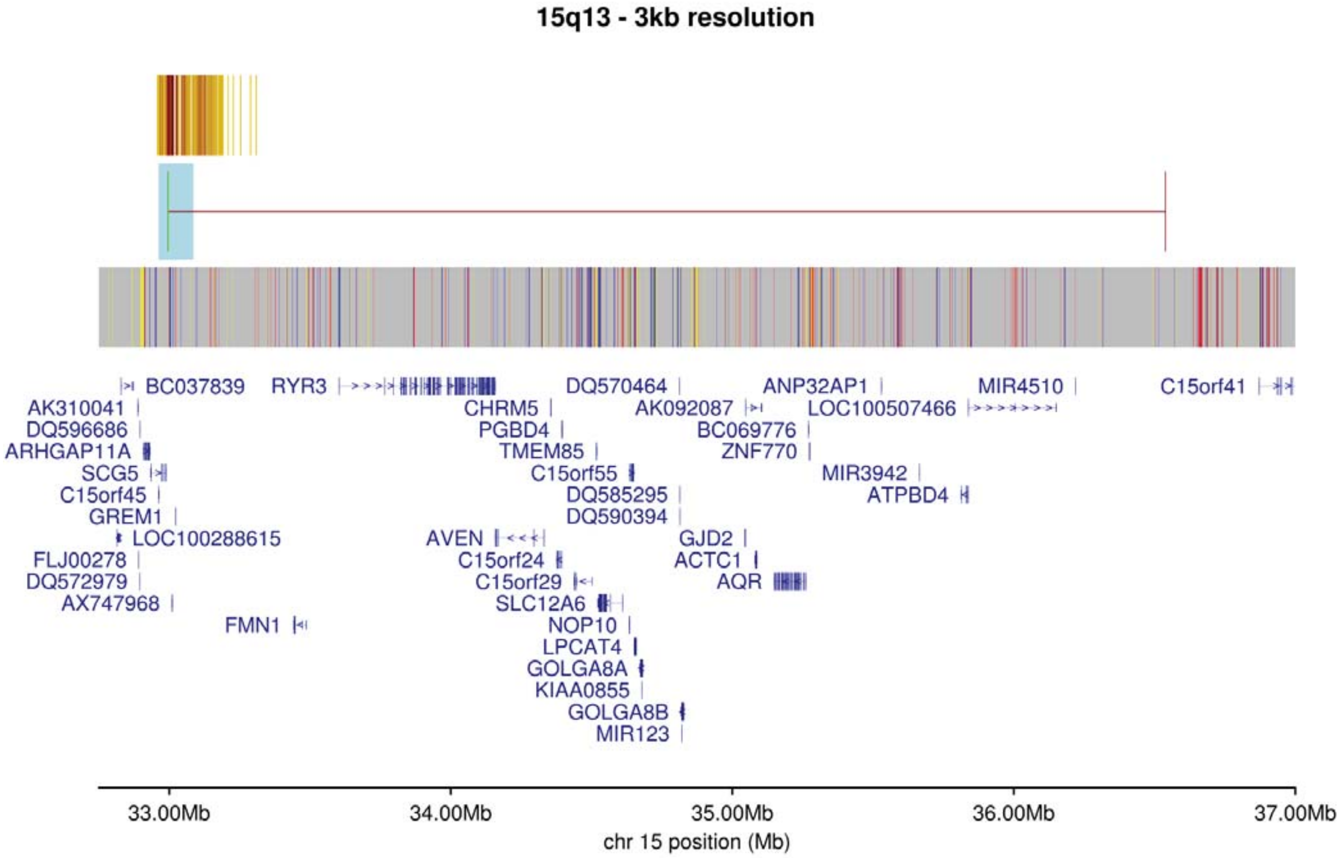

Supplementary Figure 19: Close-*cis* (+/- 5Mb) annotation of significant chromatin interactions at 15q13 at 3kb resolution. Please refer to the legend from Figure 2.

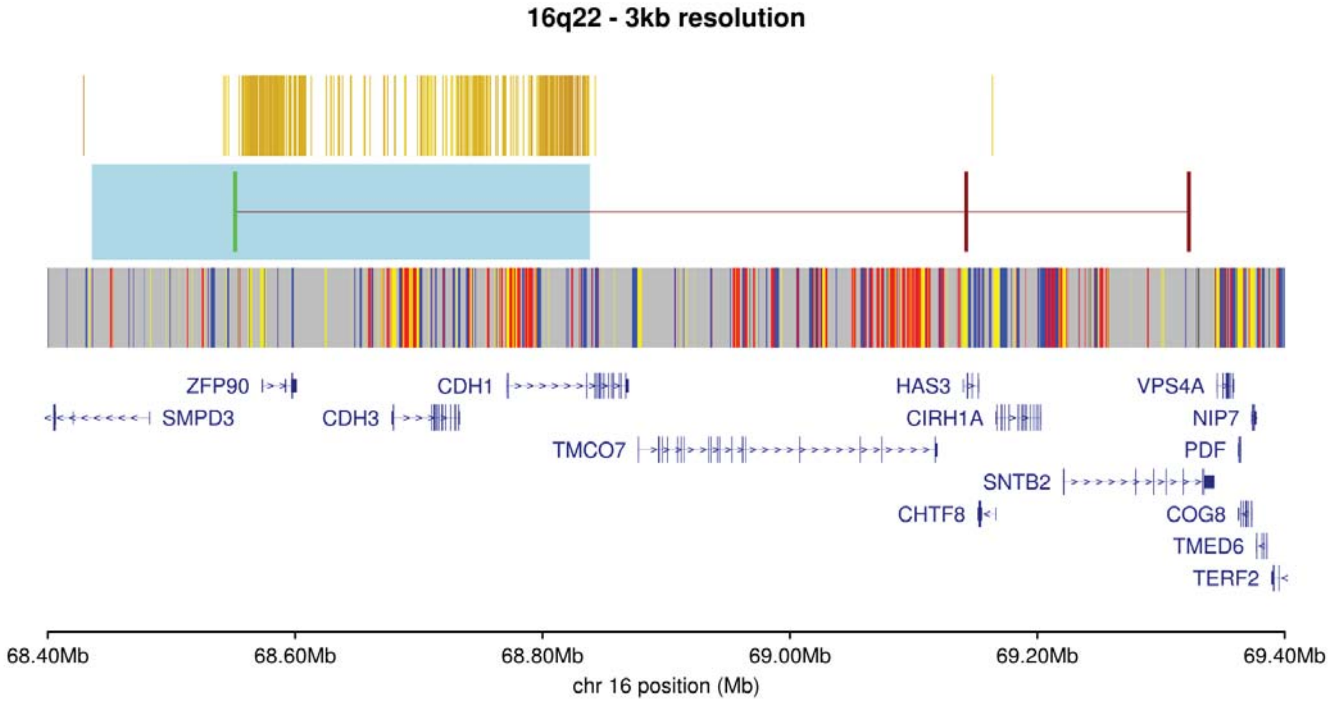

Supplementary Figure 20: Close-*cis* (+/- 5Mb) annotation of significant chromatin interactions at 16q22 at 3kb resolution. Please refer to the legend from Figure 2.

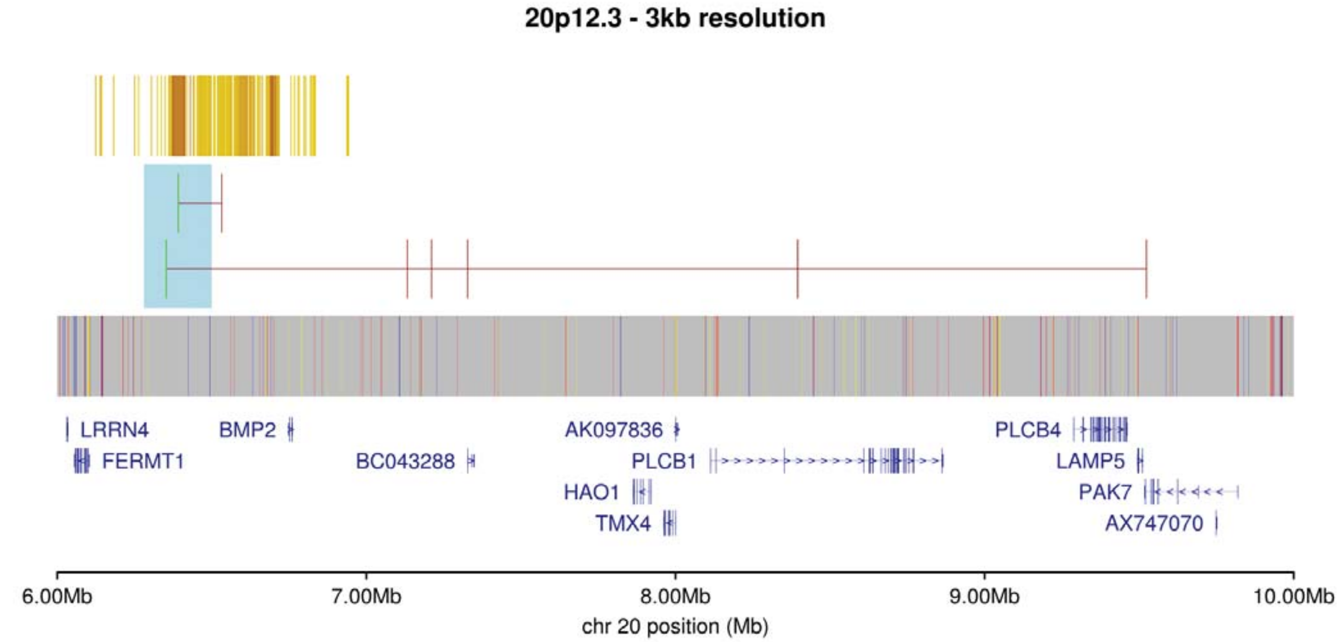

Supplementary Figure 21: Close-*cis* (+/- 5Mb) annotation of significant chromatin interactions at 20p12.3 at 3kb resolution. Please refer to the legend from Figure 2.

20q13.33 - 3kb resolution

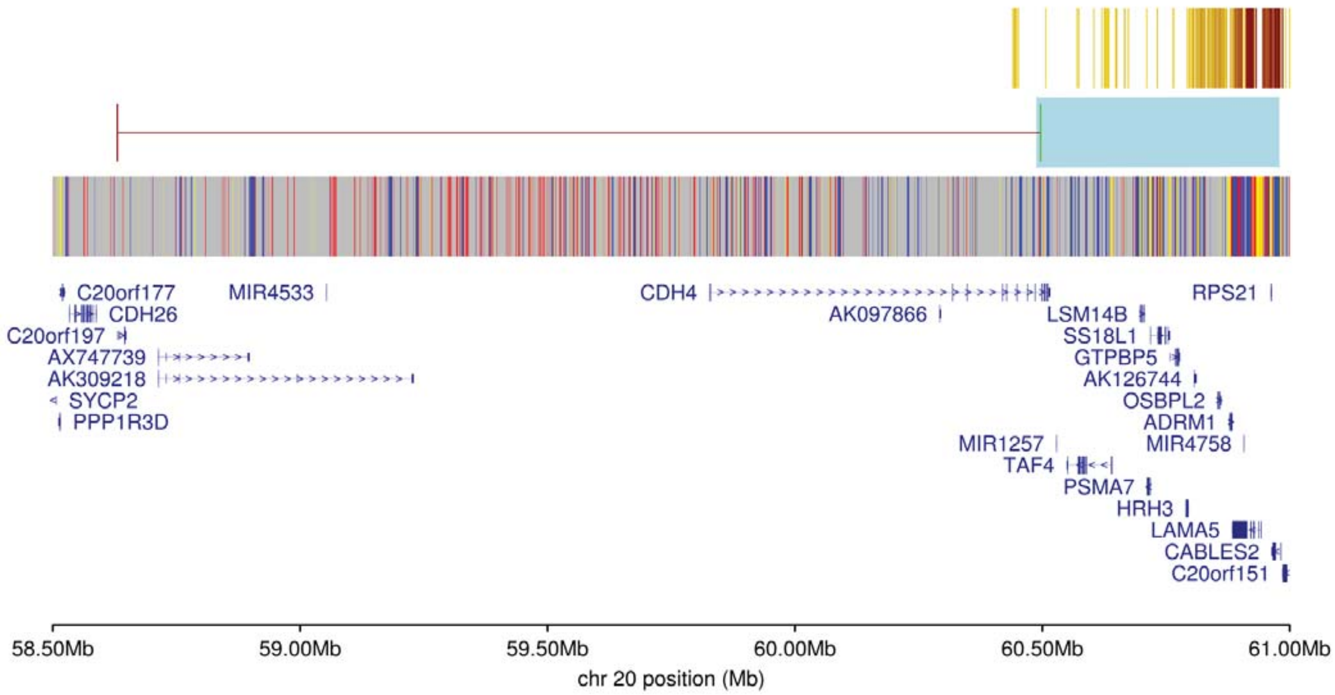

Supplementary Figure 22: Close-*cis* (+/- 5Mb) annotation of significant chromatin interactions at 20q13.33 at 3kb resolution. Please refer to the legend from Figure 2.

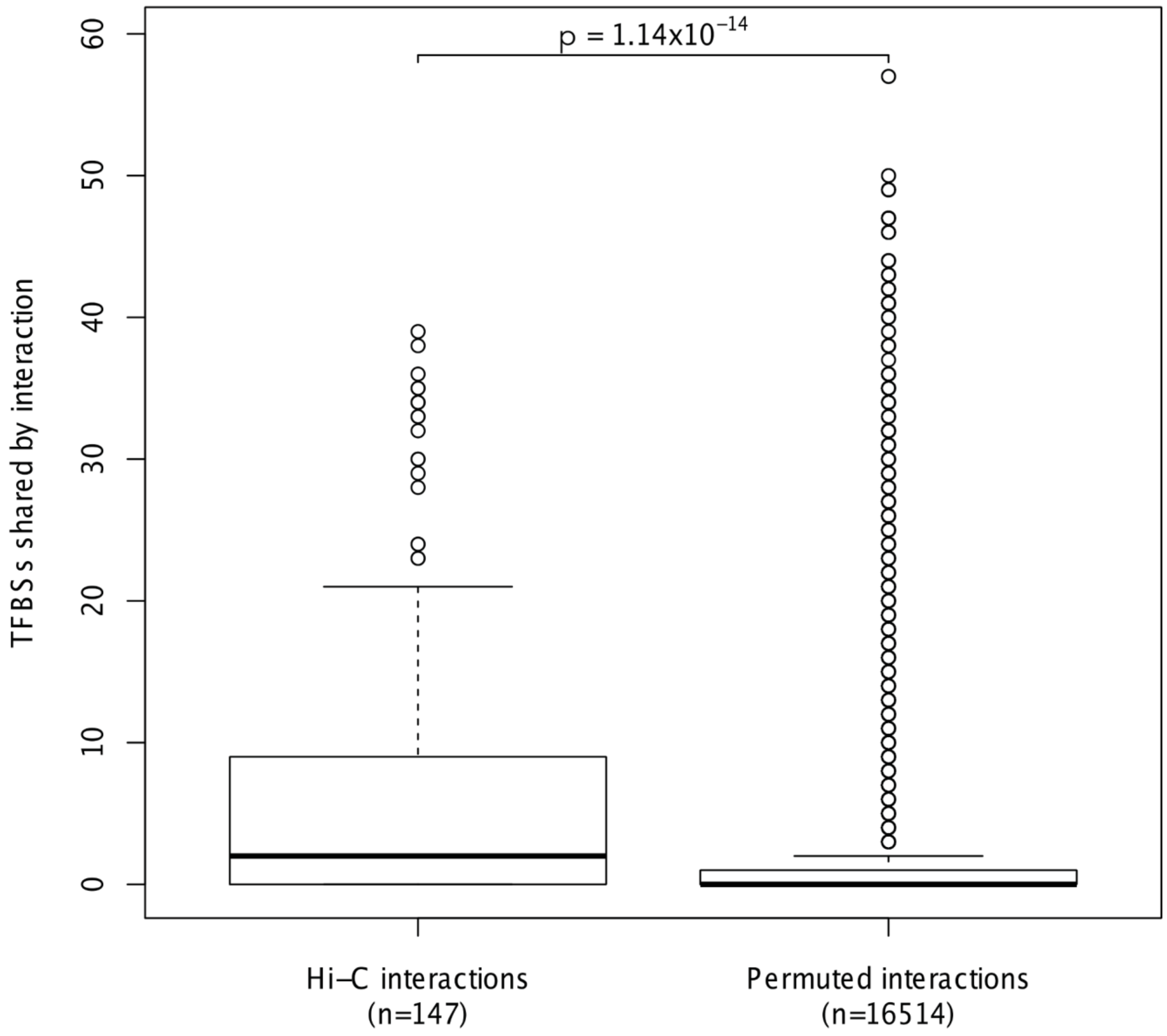

Supplementary Figure 23: Hi-C chromatin interactions are significantly enriched for shared TFs (p=1.14x10^{-14}; Mann-Whitney U test). Shown are a box plots depicting the number of TFs shared by chromatin interaction pairs compared with a permutated control set.

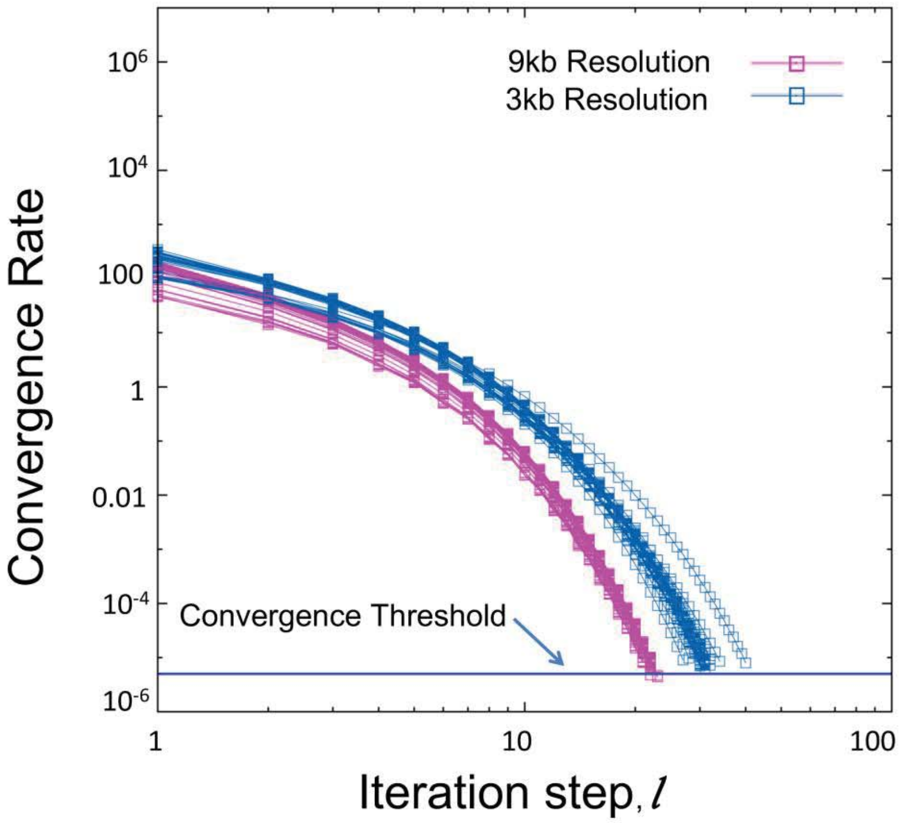

Supplementary Figure 24: Convergence rate of Supplementary Equation 1, for two different effective resolution values, as a function of the iteration step, l. The normalization procedure is based on principles previously described by Imakaev *et al*.[1], adapted for cHi-C. Shown are convergence rates of the normalization procedure (Supplementary Equation 1) as a function of the iteration step l. Convergence of the algorithm across all fifteen enriched regions was achieved for maximally possible resolutions of 9kb genome-wide (magenta line) and 3kb for close-cis (blue line). The convergence rate for N bins is defined by $\sum_{i=1}^{N} |\tilde{w}_i - 1|$, where the weights $\tilde{w}_i$, (Supplementary Equation 1) converge under iteration to unity (*i.e*. correspond to the unbiased state).

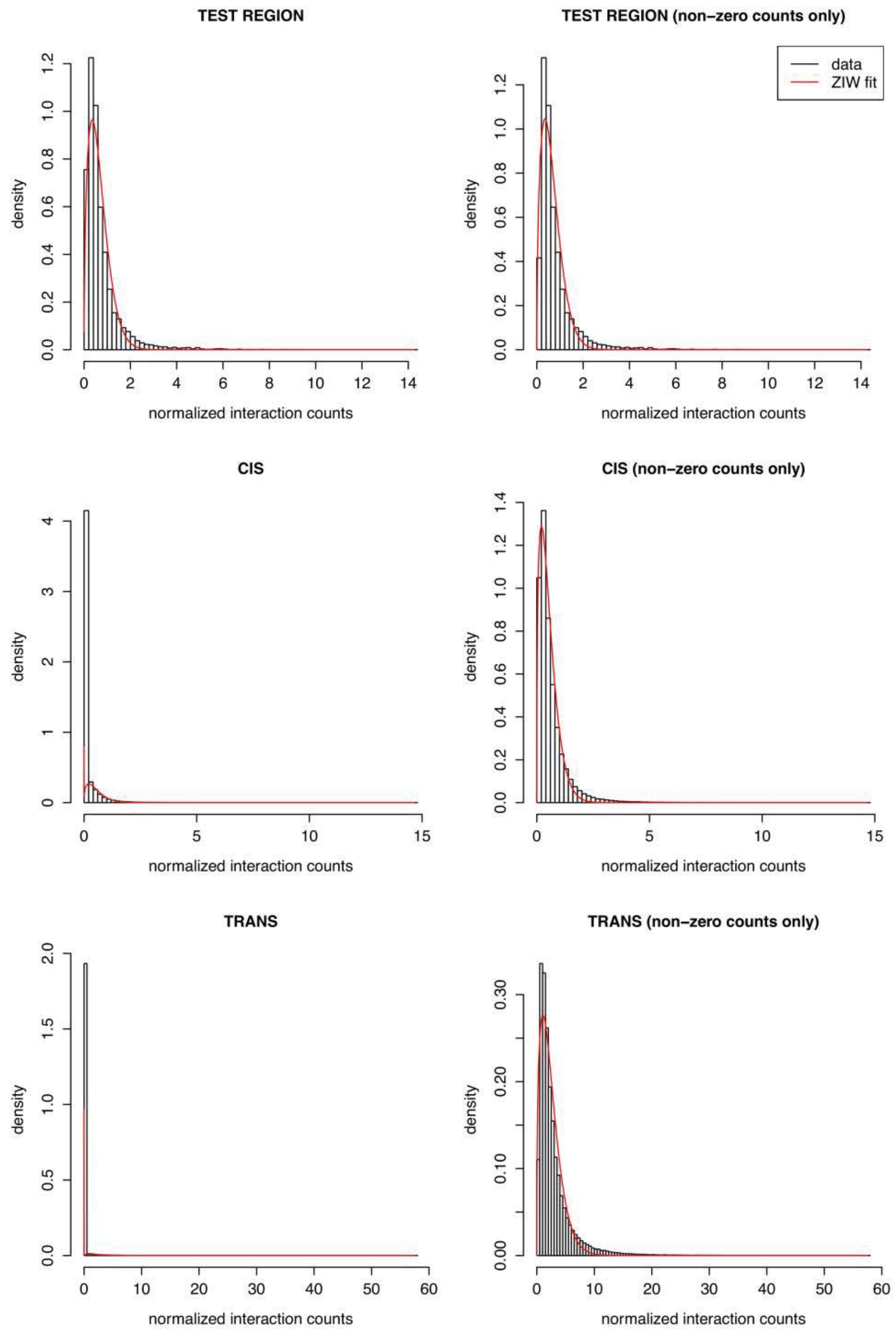

Supplementary Figure 25: Statistical analysis of cHi-C contacts. Bar plot shows density of observed bias-normalized contact frequencies. A zero-inflated Weibull distribution is fitted to the data (red line).

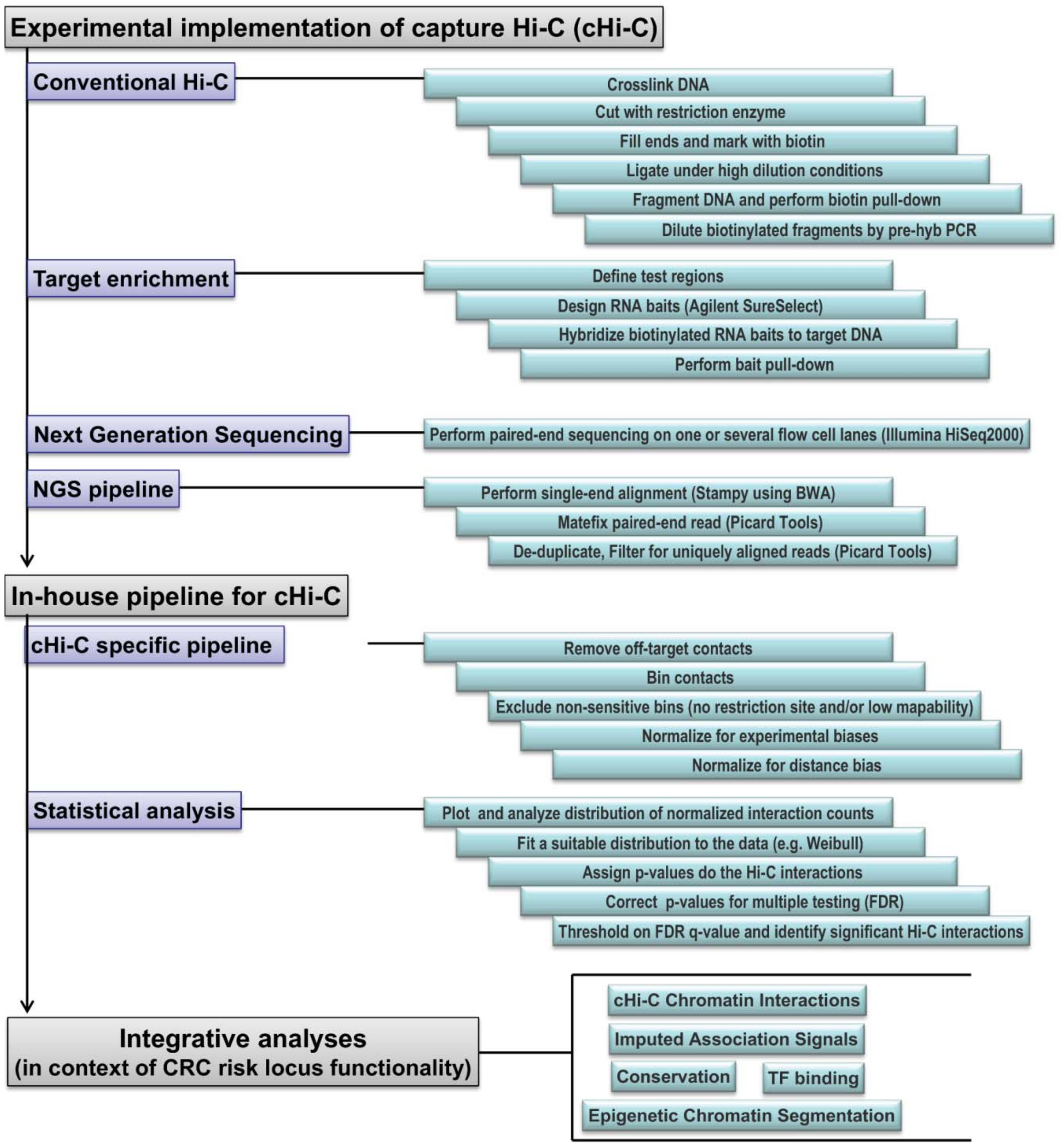

Supplementary Figure 26: Overview of cHi-C

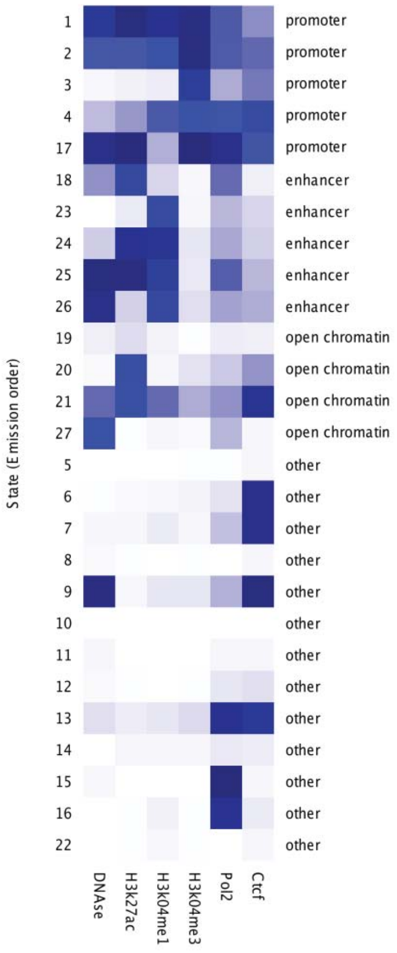

Supplementary Figure 27: Emission plot of a 27 state ChromHMM model on HCT116. Input data sets contained information on Ctcf and Pol2 binding, H3k04me3, H3k04me1 and H3k27ac modifications as well as DNAseI hypersensitivity. Dark blue squares denotes the presence, white squares the absence of specific markers.

Supplementary Tables

Supplementary Table 1: Measurement of target enrichment efficiency. a. Using enrichment factors comparing the ratios of on-target read pairs to raw reads for LS174T, Colo205, LoVo to the conventional Hi-C GM06990 library (from Lieberman-Aiden et al., Science, 2009). Size-matched random subsets were generated for LS174T, LoVo and Colo205 to achieve comparability with the full GM06990 library (30,009,111 read pairs). Size-matching of input NGS data is essential for this comparison, since PCR duplicates do not increase linearly with increasing library size (see Supplementary Table 7 for statistics on full cHi-C libraries). **b.** Using fold enrichment of on-target over off-target read pairs for each of LS174T, Colo205, LoVo.

**a.**	**LS174T**						**Colo205**						**LoVo**						**GM06990**	
	**random set 1**		**random set 2**		**random set 3**		**random set 1**		**random set 2**		**random set 3**		**random set 1**		**random set 2**		**random set 3**		**reference set**	
	**reads**	**%**	**reads**	**%**	**reads**	**%**	**reads**	**%**	**reads**	**%**	**reads**	**%**	**reads**	**%**	**reads**	**%**	**reads**	**%**	**reads**	**%**
**raw read pairs**	30,009,111	100	30,009,111	100	30,009,111	100	30,009,111	100	30,009,111	100	30,009,111	100	30,009,111	100	30,009,111	100	30,009,111	100	30,009,111	100
**de-duplicated read pairs**	21,336,066	71	20,891,233	70	22,282,895	74	17,022,076	57	16,538,405	55	16,578,142	55	19,658,965	66	18,540,775	62	18,887,952	63	29,386,668	98
**uniquely mapped read pairs**	14,237,647	47	11,540,806	38	13,404,226	45	11,815,303	39	11,815,708	39	11,769,189	39	11,676,470	39	10,473,343	35	11,668,651	39	16,414,255	55
**bona-fide Hi-C contacts**	8,713,569	29	8,826,047	29	9,080,119	30	7,273,262	24	7,287,952	24	7,264,201	24	7,556,671	25	7,875,353	26	7,551,514	25	10,628,590	35
**on-target (enriched regions)**	4,666,013	15.5	4,382,885	14.6	4,616,643	15.4	5,254,680	17.5	5,730,979	19	5,707,770	19	3,773,497	12.6	4,123,731	13.7	3,997,104	13.3	28,446	0.09
**enrichment factor**	**164**		**154**		**162**		**185**		**201**		**201**		**133**		**145**		**141**			
**mean enrichment factor: ~165**																				

**b.**	**LS147T**	**Colo205**	**LoVo**
**bona-fide Hi-C contacts**	81,805,106	25,826,005	37,487,979
**off-target (background) contacts**	47,089,250	8,015,333	24,031,485
**on-target (cHi-C) contacts**	34,715,856	17,810,672	13,456,494
**% on-target**	42%	69%	36%
**average number of reads per 9kb bin off-target**	137	23	70
**average number of reads per 9kb bin on-target**	68,204	34,991	26,437
**fold enrichment (on-target over off-target)**	**498**	**1,502**	**378**

Supplementary Table 2: Test regions defined for CRC risk locus sequence capture. Genomic positions are based on GRCh37/hg19 human genome assembly

tagSNP	Target	Chromosome	Start (bps)	End (bps)	Size (kb)
rs6687758	ENR_1	1q41	222,055,757	222,228,891	173
rs10936599	ENR_2	3q26.2	169,422,725	169,554,264	132
rs16892766	ENR_3	8q23.1	117,596,426	117,815,078	219
rs6983267	ENR_4	8q24.21	128,106,784	129,224,599	1118
rs10795668	ENR_5	10p14	8,636,919	8,762,195	125
rs3802842	ENR_6	11q23	111,119,694	111,215,664	96
rs7136702/rs11169552	ENR_7	12q13	50,433,234	51,320,290	887
rs4444235/rs1957636	ENR_8	14q22.2	54,383,945	54,802,844	419
rs11632715/rs16969681	ENR_9	15q13	32,968,831	33,087,705	119
rs9929218	ENR_10	16q22.1	68,433,045	68,839,302	406
rs4939827	ENR_11	18q21.1	46,371,993	46,463,136	91
rs10411210	ENR_12	19q13.1	33,513,710	33,670,981	157
rs961253	ENR_13	20p12.3	6,281,204	6,501,619	220
rs4813802	ENR_14	20p12.3	6,690,101	6,716,097	26
rs4925386	ENR_15	20q13.33	60,486,203	60,981,155	495
				**Total**	**4683**

Supplementary Table 3: Validation of cHi-C interactions by co-localization analysis using interphase FISH. Locations of tested interactions are listed in Supplementary Data 1.

							Co-localization		
**Probe set**	**Interaction**	**Distance (Mb)**	**Probe_1**	**Probe_2**	**Probe_3**	**n**[1]	**Hi-C**	**Control**	**p-value**[2]
cis_1	9kb_contact_8	23.5	RP11-990E14	RP11-1131E12	RP11-217F11	424	4.48%	1.42%	1.30E-02
cis_2	9kb_contact_39	80.8	RP11-63M17	RP11-904N7	RP11-507I5	6,080	2.30%	0.44%	8.00E-20
cis_3	9kb_contact_129	65.8	RP11-96E12	RP11-1083M5	RP11-369O17	3,318	6.24%	4.25%	3.30E-04
cis_4	9kb_contact_38	45.1	RP11-3M16	RP11-727O22	RP11-59I15	2,760	2.39%	0.80%	2.40E-06
cis_5	9kb_contact_135	26.9	RP11-1058N17	RP11-60G3	RP11-643C20	2,666	10.05%	5.14%	1.30E-11
cis_6	9kb_contact_94	24.1	RP11-99J13	RP11-21F12	RP11-26M6	#	#	#	#
cis_7	9kb_contact_31	16.9	RP11-76P21	RP11-702B10	RP11-790H21	*	*	*	*
trans_1	9kb_contact_9	trans	RP11-194D7	RP11-165L17	RP11-382A12	*	*	*	*
trans_2	9kb_contact_25	trans	RP11-346N3	RP11-465J16	RP11-557N1	*	*	*	*
trans_3	9kb_contact_26	trans	RP11-76P21	RP5-1108D11	RP11-122O1	*	*	*	*
trans_4	9kb_contact_67	trans	RP11-77C15	RP11-264H23	RP11-95J1	3,882	1.26%	0.46%	1.80E-04
trans_5	9kb_contact_70	trans	RP11-99J13	RP11-1077I8	RP11-780D7	*	*	*	*
trans_6	9kb_contact_69	trans	RP11-99J13	RP11-161L13	RP11-455A1	5,062	0.87%	0.47%	2.00E-02
trans_7	9kb_contact_143	trans	RP11-15D8	RP11-134J10	RP11-305B23	*	*	*	*

[1] Number of total alleles analyzed after image processing
[2] Fisher's exact test
Minimum number of alleles after image processing (n>400) not achieved
* One or several probes unspecific in metaphase FISH

Supplementary Table 4: Fisher's exact test on enrichment of significantly interacting cHi-C test bins at sites of high CRC risk association

	non-interacting test bins	interacting test bins	total	
**overlap with low association p-value**	65	23	88	
**no overlap**	240	38	278	
**total**	305	61	366	**p=8.54E-03**

Supplementary Table 5: Integration of top-associated SNPs at candidate causative elements with associated evolutionary conservation, transcription factor (TF) binding and chromatin state. Top ten associations for each locus are detailed if localised within a significantly interacting cHi-c test bin. For evolutionary conservation phastCons and GERP scores are shown (see "Methods"). At SNPs, the total number of TFs found to bind in LoVo cells is listed. Further, corresponding cHi-C test bins are shown, referring to the detailed list of TFs shared by the test bin at the SNP and the cHi-C interactor bin provided in Supplementary Table 11. Chromatin states (4-state-scheme, see Supplementary Table 10) at sites of SNPs are listed.

risk locus	SNP	chr	position	association p-value[1]	strongest association in region	phastCons score	GERP score	total number of TF	cHi-C test bin	chromatin state
**3q26.2**	rs2293607	3	169482335	3.47E-04	4.64E-05	0.00	-6.81	50	9kb_contact_8	promoter
	rs12638862	3	169477506	4.41E-03		0.00	-3.58	4	9kb_contact_8	other
	rs12630450	3	169480204	5.36E-03		0.00	-0.799	1	9kb_contact_8	other
	rs12696304	3	169481271	5.78E-03		0.00	-3.47	42	9kb_contact_8	promoter
**8q23.1**	rs16888611	8	117642990	4.02E-07	2.51E-09	0.00	0.747	0	N/A	other
	rs28535528	8	117642428	4.12E-07		0.00	-1.17	0	N/A	other
	rs16892766	8	117630683	6.17E-07		1.00	6.07	0	N/A	other
	rs16888589	8	117635602	8.09E-07		0.00	-1.05	0	N/A	other
	rs11986063	8	117640315	1.52E-06		0.00	1.18	0	N/A	other
	rs1370095	8	117642716	7.62E-03		0.00	1.36	0	N/A	other
	rs10111334	8	117643271	8.60E-03		0.01	2.83	0	N/A	other
	rs9650067	8	117643728	9.52E-03		1.00	3.68	0	N/A	other
	rs9650068	8	117643773	9.92E-03		0.92	1.34	0	N/A	other
	rs7834164	8	117644462	1.89E-02		0.00	-0.745	0	N/A	other
**8q24.21**	rs6983267	8	128413305	1.64E-12	1.64E-12	1.00	5.31	64	9kb_contacts_16-19	enhancer
	rs12682374	8	128410948	1.64E-12		0.00	-2.11	5	9kb_contacts_16-19	other
	rs11997201	8	128415734	2.46E-12		0.00	1.08	27	9kb_contacts_16-19	enhancer
	rs7013278	8	128414892	2.50E-12		0.00	-2.96	31	9kb_contacts_16-19	enhancer
	rs10956368	8	128423650	2.80E-12		0.00	-0.321	1	9kb_contacts_20-22	other
	rs10505474	8	128417504	3.22E-12		0.00	0.276	4	9kb_contacts_16-19	other

	rs6983549	8	128420712	3.26E-12		0.00	-4.12	1	9kb_contacts_20-22	other
	rs10808556	8	128413147	3.30E-12		0.00	-1.38	64	9kb_contacts_16-19	enhancer
	rs7014346	8	128424792	3.67E-12		0.00	2.24	1	9kb_contacts_20-22	other
	rs7837328	8	128423127	3.85E-12		0.00	-0.732	1	9kb_contacts_20-22	other
**10p14**	rs12777423	10	8729712	8.09E-11	9.75E-12	0.00	-0.47	0	N/A	other
	rs7894531	10	8734761	2.96E-10		0.00	-5.93	0	N/A	other
	rs12358150	10	8735744	3.17E-10		0.12	0.199	0	N/A	other
	rs10905449	10	8728419	5.33E-06		0.00	-0.31	0	N/A	other
	rs11255831	10	8729909	8.81E-06		0.00	-1.08	0	N/A	other
	rs11255835	10	8732887	9.65E-06		0.00	-0.557	1	9kb_contact_25	other
	rs7912704	10	8731175	9.89E-06		0.00	2.76	2	9kb_contact_25	other
	rs7912831	10	8731255	1.04E-05		0.00	2.56	1	9kb_contact_25	other
	rs1537603	10	8734295	1.38E-05		0.00	0	0	N/A	other
	rs11255834	10	8732235	8.92E-04		0.00	-1.02	0	N/A	other
**11q23**	rs11213823	11	111169990	1.18E-06	7.20E-07	0.00	1.86	31	9kb_contacts_30-31	other
	rs10789822	11	111162691	1.23E-06		0.00	0.545	0	N/A	other
	rs7103178	11	111165009	1.31E-06		0.00	1.07	1	9kb_contacts_30-31	other
	rs12296076	11	111166504	1.49E-06		0.00	-4.28	0	N/A	other
	rs7944895	11	111167776	1.76E-06		0.00	-0.326	0	N/A	other
	rs6589218	11	111167557	1.85E-06		0.00	-0.00518	1	9kb_contacts_30-31	other
	rs10891246	11	111170540	2.47E-06		0.00	-5.67	33	9kb_contacts_30-31	other
	rs7105857	11	111170744	2.47E-06		0.00	1.21	25	9kb_contacts_30-31	other
**12q13**	rs3184122	12	50570127	9.41E-08	2.56E-09	0.00	-0.926	14	9kb_contact_39	open chromatin
	rs34245511	12	50573433	1.30E-07		0.01	0	0	N/A	other
	rs9364	12	50570519	1.54E-07		0.00	-2.74	5	9kb_contact_39	open chromatin
	rs12424335	12	50574763	1.54E-07		0.00	-0.178	0	N/A	open chromatin
	rs11169281	12	50529314	1.93E-07		0.00	-7.93	4	9kb_contact_38	other

	rs11169278	12	50525787	2.06E-07		0.01	0.494	0	N/A	other
	rs1554845	12	50523504	2.12E-07		0.00	1.61	2	9kb_contact_38	other
	rs34309034	12	50573500	2.13E-07		0.01	0	0	N/A	other
	rs11169282	12	50529971	2.29E-07		0.09	0.392	4	9kb_contact_38	other
	rs3741562	12	50529736	2.37E-07		1.00	4.63	4	9kb_contact_38	other
**14q22.2**	rs35107139	14	54419106	3.53E-05	3.53E-05	0.00	2.39	41	9kb_contacts_52-59	enhancer
	rs2071047	14	54418411	1.28E-04		0.00	0.216	33	9kb_contacts_52-59	enhancer
	rs10130587	14	54419110	2.35E-04		0.01	3.3	41	9kb_contacts_52-59	enhancer
	rs4444235	14	54410919	3.34E-04		0.00	-2.52	14	9kb_contacts_48-51	open chromatin
	rs1953743	14	54652479	4.69E-04		0.94	4.03	0	N/A	other
	rs12896913	14	54649293	4.95E-04		0.12	0.225	23	9kb_contact_116	enhancer
	rs11623717	14	54414132	5.02E-04		0.00	-2.77	20	9kb_contacts_52-59	enhancer
	rs36020508	14	54647592	5.58E-04		0.00	-6.15	13	9kb_contacts_107-115	enhancer
	rs12893484	14	54414738	5.98E-04		1.00	4.04	35	9kb_contacts_52-59	enhancer
	rs11157993	14	54411696	6.54E-04		0.00	-3.95	5	9kb_contacts_48-51	open chromatin
**15q13**	rs1406389	15	33009478	7.83E-11	7.83E-11	0.00	1.2	1	9kb_contact_129	other
	rs2293582	15	33010412	8.08E-11		0.00	-0.884	2	9kb_contact_129	other
	rs2293581	15	33010736	8.20E-11		0.02	2.46	3	9kb_contact_129	other
	rs73376930	15	33012502	9.92E-11		0.00	-7.89	0	N/A	other
	rs79207432	15	33011697	1.68E-10		0.00	-1.04	2	9kb_contact_129	other
	rs1919364	15	33009574	1.87E-06		0.09	2.08	1	9kb_contact_129	other
	rs11635984	15	33012232	4.13E-06		0.00	1.17	0	N/A	other
	rs72715291	15	33009231	1.58E-02		0.01	3.36	0	N/A	other
**18q21**	rs4939567	18	46451873	6.60E-17	6.60E-17	0.00	0.0513	0	N/A	enhancer
	rs11874392	18	46453156	7.59E-17		0.25	-1.57	1	9kb_contact_135	other
	rs2337113	18	46452327	9.22E-17		0.02	0.225	1	9kb_contact_135	enhancer
	rs7226855	18	46454048	1.79E-16		0.00	-5.95	2	9kb_contact_135	other

	rs4939827	18	46453463	7.31E-16		0.00	1.32	0	N/A	other
	rs34007497	18	46451073	1.01E-15		0.00	0.92	3	9kb_contact_135	enhancer
	rs8085824	18	46449111	3.69E-15		0.97	1.41	1	9kb_contact_135	enhancer
	rs12953717	18	46453929	4.29E-15		0.00	1.01	2	9kb_contact_135	other
	rs58920878	18	46449565	4.44E-14		0.00	-0.687	4	9kb_contact_135	promoter
	rs12956924	18	46451146	8.28E-09		0.00	-4.49	3	9kb_contact_135	enhancer
**20p12.3**	rs35469553	20	6386438	8.44E-06	3.38E-08	0.01	-0.384	0	N/A	other
	rs437708	20	6378673	8.53E-06		0.00	-1.38	0	N/A	other
	rs355528	20	6376017	8.90E-06		0.00	-0.0979	1	9kb_contact_143	other
	rs36026248	20	6382002	9.12E-06		0.01	-0.388	0	N/A	other
	rs438030	20	6378672	9.14E-06		0.00	0.69	0	N/A	other
	rs654433	20	6380022	1.06E-05		0.00	-0.585	0	N/A	other
	rs189583	20	6376457	1.10E-05		0.85	2.27	0	N/A	other
	rs966816	20	6376481	1.10E-05		0.00	-1.05	0	N/A	other

[1] **F**ixed-effects meta-analysis of Cochran-Armitage trend test results

Supplementary Table 6: Number of interactor bins (genome wide, 9kb resolution) overlapping regulatory elements (enhancer, promoter) in HCT116 (highlighted by shaded grey cells) and non-CRC cell lines. *p*-values for pairwise overlap significance compared to the reference HCT116 cell line are computed using Fisher's exact test. The overlap with promoter regions is highly tissue specific for the colorectal cancer cell line with all 9 *p*-values well below 0.05. The overlap with enhancer regions also shows some evidence for tissue specificity with an enrichment in low p-values (1 out of 9 *p*-values expected to be < 0.11 under the null hypothesis of no association between the overlap count and the cell line type; we count 6 *p*-values < 0.11).

	**HCT116** (Colorectal carcinoma)	**GM12878** (Lymphoblastoid)	**H1hESC** (Embryonic stem cell)	**HEPG2** (Hepatocellular carcinoma)	**HMEC** (Mammary epithelial cell)	**HSMM** (Skeletal muscle myoblast)	**HUVEC** (Umbilical vein endothelial cell)	**K562** (Chronic myelogenous leukemia)	**NHEK** (Human epidermal keratinocyte)	**NHLF** (Human lung fibroblast)
**ENHANCER REGIONS**										
overlap	48	30	30	36	43	38	40	17	35	29
non-overlap	43	61	61	55	48	53	51	74	56	62
p		0.01*	0.01*	0.10	0.55	0.18	0.29	2.54×10^{-6}*	0.07	6.74×10^{-3}*
**PROMOTER REGIONS**										
overlap	30	9	11	12	7	10	11	8	9	9
non-overlap	61	82	80	79	84	81	80	83	82	82
p		2.35×10^{-4}*	1.22×10^{-3}*	2.52×10^{-3}*	3.23×10^{-5}*	5.57×10^{-4}*	1.22×10^{-3}*	9.14×10^{-5}*	2.35×10^{-4}*	2.35×10^{-4}*

* $p < 0.05$

ChromHMM-based chromatin segmentation for non-CRC cell lines listed in the table were downloaded from the ENCODE database; http://hgdownload-test.cse.ucsc.edu/goldenPath/hg19/encodeDCC/wgEncodeBroadHmm/

Supplementary Table 7: Summary statistics on NGS read mapping, NGS filtering, Hi-C specific filtering and target enrichment for the three CRC cell lines LS174T, LoVo and Colo205

	**LS147T**		**LoVo**		**Colo205**	
	**read counts**	**% remaining**	**read counts**	**% remaining**	**read counts**	**% remaining**
**raw reads pairs**	831,276,898	100	738,928,113	100	832,973,525	100
**de-duplicated read pairs**	312,994,947	38	145,793,339	20	87,427,635	10
**uniquely mapped read pairs**	124,012,010	15	53,340,837	7	33,523,776	4
**bona-fide Hi-C contacts**	81,805,106	10	37,487,979	5	25,826,005	3
**within enriched regions**	34,715,856	4	13,456,494	2	17,810,672	2

Supplementary Table 8: Primers for 4C-seq (designed using the 4C primer design software: http://mnlab.uchicago.edu/4Cpd/help.html)

Hi-C bin	primary restriction enzyme	secondary restriction enzyme	Reading primer	Non reading primer
chr3:169440000-169443000	*HindIII*	*Csp6I*	AATGATACGGCGACCACCGAACACTCTTTCCCTACACG ACGCTCTTCCGATCTACATTTGGCCACAAGCTT	CAAGCAGAAGACGGCATACGACCAGGTGAG ATATTTGGCCATT
chr3:169458000-169461000	*HindIII*	*DpnII*	AATGATACGGCGACCACCGAACACTCTTTCCCTACACG ACGCTCTTCCGATCTCTTTCGCAGTGGAAGCTT	CAAGCAGAAGACGGCATACGAAGCAGAGAC AGGTTTCGCC
chr3:169461000-169464000	*HindIII*	*DpnII*	AATGATACGGCGACCACCGAACACTCTTTCCCTACACG ACGCTCTTCCGATCTCGAGGTTCTCGCAAGCTT	CAAGCAGAAGACGGCATACGAGAATTTCGGC CACCAAAT
chr3:169488000-169491000	*HindIII*	*NlaIII*	AATGATACGGCGACCACCGAACACTCTTTCCCTACACG ACGCTCTTCCGATCTAAGTGCGTGCAGATTCAAGCTT	CAAGCAGAAGACGGCATACGAGCTTCCGAA GACCAAGCAG
chr8:128409000-128412000	*HindIII*	*NlaIII*	AATGATACGGCGACCACCGAACACTCTTTCCCTACACG ACGCTCTTCCGATCTCCAGGCTAAGTCACAAGCTT	CAAGCAGAAGACGGCATACGATGACCGACTA CCTGAGCATG
chr8:128412000-128415000	*HindIII*	*Csp6I*	AATGATACGGCGACCACCGAACACTCTTTCCCTACACG ACGCTCTTCCGATCTCAATGCATTAGATGAAGCTT	CAAGCAGAAGACGGCATACGAAACTGGTATT ACAGCCTGCT
chr11:111165000-111168000	*HindIII*	*DpnII*	AATGATACGGCGACCACCGAACACTCTTTCCCTACACG ACGCTCTTCCGATCTGACTGTATTACCAAGCTT	CAAGCAGAAGACGGCATACGAGGATTTCTGA GGTCAATGGC
chr14:54795000-54798000	*HindIII*	*NlaIII*	AATGATACGGCGACCACCGAACACTCTTTCCCTACACG ACGCTCTTCCGATCTTTGTCAGGGAGTTAAGCTT	CAAGCAGAAGACGGCATACGACTGATGCTAA GAGGCACTAT
chr14:54798000-54801000	*HindIII*	*DpnII*	AATGATACGGCGACCACCGAACACTCTTTCCCTACACG ACGCTCTTCCGATCTACCTAACCTAATCAAGCTT	CAAGCAGAAGACGGCATACGAACGATGTCTC CGAAAGATGA
chr14:54009000-54012000	*HindIII*	*DpnII*	AATGATACGGCGACCACCGAACACTCTTTCCCTACACG ACGCTCTTCCGATCTTCTGACCTTTAGTAAGCTT	CAAGCAGAAGACGGCATACGATGGCTTGATT TGCTACATTC
chr16:68550000-68553000	*HindIII*	*NlaIII*	AATGATACGGCGACCACCGAACACTCTTTCCCTACACG ACGCTCTTCCGATCTGAACAGCCACACCAGCAAGCTT	CAAGCAGAAGACGGCATACGACATTTTGCTG GGCGCCGT

Supplementary Table 9: Chip-Seq and DNAse-Seq experiments on HCT116 downloaded from the ENCODE database for generating a CRC specific epigenetic chromatin segmentation using ChromHMM

**Type**	**Mark**	**Source**	**Replicate**	**File:** http://hgdownload.cse.ucsc.edu/goldenPath/hg19/encodeDCC/
Dnase	Dnase	Uw	Rep1	wgEncodeUwDnase/wgEncodeUwDnaseHct116AlnRep1.bam
Dnase	Dnase	Uw	Rep2	wgEncodeUwDnase/wgEncodeUwDnaseHct116AlnRep2.bam
Histone	Input	Uw	Rep1	wgEncodeUwHistone/wgEncodeUwHistoneHct116InputStdAlnRep1.bam
Histone	Input	Sydh	Rep1	wgEncodeSydhHistone/wgEncodeSydhHistoneHct116InputUcdAlnRep1.bam
Histone	H3k04me1	Sydh	Rep2	wgEncodeSydhHistone/wgEncodeSydhHistoneHct116H3k04me1UcdAlnRep2.bam
Histone	H3k27ac	Sydh	Rep1	wgEncodeSydhHistone/wgEncodeSydhHistoneHct116H3k27acUcdAlnRep1.bam
Histone	H3k27ac	Sydh	Rep2	wgEncodeSydhHistone/wgEncodeSydhHistoneHct116H3k27acUcdAlnRep2.bam
Histone	H3k4me3	Uw	Rep1	wgEncodeUwHistone/wgEncodeUwHistoneHct116H3k4me3StdAlnRep1.bam
Histone	H3k4me3	Uw	Rep2	wgEncodeUwHistone/wgEncodeUwHistoneHct116H3k4me3StdAlnRep2.bam
Tfbs	Input	Uw	Rep1	wgEncodeUwTfbs/wgEncodeUwTfbsHct116InputStdAlnRep1.bam
Tfbs	Input	Sydh	Rep1	wgEncodeSydhTfbs/wgEncodeSydhTfbsHct116InputUcdAlnRep1.bam
Tfbs	Ctcf	Haib	Rep1	wgEncodeHaibTfbs/wgEncodeHaibTfbsHct116CtcfcV0422111AlnRep1.bam
Tfbs	Ctcf	Haib	Rep2	wgEncodeHaibTfbs/wgEncodeHaibTfbsHct116CtcfcV0422111AlnRep2.bam
Tfbs	Pol2	Sydh	Rep1	wgEncodeSydhTfbs/wgEncodeSydhTfbsHct116Pol2UcdAlnRep1.bam
Tfbs	Pol2	Sydh	Rep2	wgEncodeSydhTfbs/wgEncodeSydhTfbsHct116Pol2UcdAlnRep2.bam

Supplementary Table 10: Conversion of the previously published 15–state-ChromHMM-annotation to a four-state-scheme. Data from http://hgdownload.cse.ucsc.edu/goldenpath/hg19/encodeDCC/wgEncodeBroadHmm/

**15-state scheme**	**colour (RGB)**	**4-state scheme**	**colour (RGB)**
1_Active_Promoter	255,0,0	promoter	255,255,0
2_Weak_Promoter	255,105,105	promoter	255,255,0
3_Poised_Promoter	207,11,198	promoter	255,255,0
4_Strong_Enhancer	250,202,0	enhancer	255,0,0
5_Strong_Enhancer	250,202,0	enhancer	255,0,0
6_Weak_Enhancer	255,252,4	enhancer	255,0,0
7_Weak_Enhancer	255,252,4	enhancer	255,0,0
8_Insulator	10,190,254	other	190,190,190
9_Txn_Transition	0,176,80	other	190,190,190
10_Txn_Elongation	0,176,80	open_chromatin	0,0,255
11_Weak_Txn	153,255,102	open_chromatin	0,0,255
12_Repressed	127,127,127	other	190,190,190
13_Heterochrom/lo	245,245,245	other	190,190,190
14_Repetitive/CNV	245,245,245	other	190,190,190
15_Repetitive/CNV	245,245,245	other	190,190,190

Supplementary Table 11: cHi-C interactions with TFBS(s) shared between test bin and interactor bin. Coordinates of contacts are listed in Supplementary Data 1.

Hi-C contact	Transcription factor(s) shared by Hi-C contact (test bin and interactor bin)
9kb_contact_5	CTCF, RAD21, SMC1A, SMC3
9kb_contact_6	CTCF, RAD21, SMAD2, SMC1A, SMC3
9kb_contact_8	CLOCK, E2F8
9kb_contact_15	CTCF, CTCFL, RAD21, SA1, SMC1A, SMC3
9kb_contact_16	ATF5, CTCF, ELF2, LYL1, RAD21, SMC1A, SMC3, SOX9, SREBF1
9kb_contact_17	ATF5, BARHL1, BARX1, CEBPB, CUX1, DLX1, E2F7, EGR1, ELF3, ESR1, Ets-2, EVI1, FOXP1, GLI2, HOXA13, HOXB13, JUND, MED12, MED1, MYC, NIPBL, RAD21, RXRA, SMC1A, SMC3, TAL1, TEAD2, ZNF217, ZNF250, ZNF281
9kb_contact_18	ATF5, BARHL1, BARX1, CUX1, DLX1, E2F2, E2F7, EGR1, ELF2, ELF3, EP300, FOXG1, FOXP1, GLI2, GMEB1, HOXA13, HOXC6, LYL1, MED12, MED1, MNT, MYC, PATZ1, RAD21, REST, SMC1A, SMC3, TEAD2, ZNF250
9kb_contact_19	CTCF, JUND, RAD21, SMC1A, SMC3
9kb_contact_22	STAT3
9kb_contact_28	CAMTA2, ERM, Ets-2, MNT, MYC, NIPBL, RAD21, RXRA
9kb_contact_29	REST
9kb_contact_30	CTCF, HINFP, MED12, MYC, RAD21, SMAD2, SMC1A, SMC3, ZNF83
9kb_contact_33	HINFP, ZBED4
9kb_contact_37	TFDP1
9kb_contact_38	HOXA10
9kb_contact_40	E2F7, JUND, RAD21, ZNF143
9kb_contact_42	ATF5, BARHL1, CEBPB, CLOCK, DLX1, E2F7, ELF2, ERM, ESR1, Ets-2, EVI1, FOXA1, FOXG1, FOXP1, GMEB1, HOXA13, IRF1, JUN, JUND, LYL1, MED12, MED1, MNT, MYC, NFAT5, NIPBL, RAD21, REST, RXRA, SMC1A, SMC3, TAL1, TEAD2, UBTF, VEZF1, ZBTB10
9kb_contact_47	ADNP, CTCF, CTCFL, Ets-2, EVI1, HOXA13, JUND, MED12, NIPBL, RAD21, REST, RORA, SA1, SMC1A, SMC3
9kb_contact_48	E2F3, ERM, Ets-2, JUND, NIPBL, PATZ1, RAD21, SMC1A, SMC3
9kb_contact_49	NIPBL, RAD21, SMC1A, SMC3
9kb_contact_50	RAD21
9kb_contact_51	ATF1
9kb_contact_52	ADNP, E2F7, EVI1, HOXA13, PATZ1, RAD21, RFX5, RORA, SA1
9kb_contact_53	ATF5, RAD21

9kb_contact_54	ADNP, ATF5, BARHL1, CEBPB, DBP, E2F7, EVI1, FOXG1, GLI2, GLI3, GMEB1, HBP1, HIVEP1, HOXA13, HOXB13, HOXC6, LYL1, MED1, MNT, MYC, MZF1, NFAT5, NFE2L2, PATZ1, RAD21, RARG, RFX5, RORA, RXRA, SA1, TAL1, VEZF1, ZBTB10, ZNF143, ZNF250
9kb_contact_55	ADNP, ATF5, BARHL1, CEBPB, DBP, E2F7, EVI1, FOXG1, GMEB1, HIVEP1, HOXC6, LYL1, NFAT5, NFE2L2, RFX5, RORA, ZNF250
9kb_contact_56	LYL1, RXRA
9kb_contact_57	ATF5, CEBPB, DBP, E2F7, EVI1, FOXG1, GLI2, HIVEP1, HOXA1, JAZF1, LYL1, MED1, MNT, MYC, NFAT5, PATZ1, RAD21, RORA, RXRA, TAL1, TP73, VEZF1, ZBTB10, ZNF143
9kb_contact_58	ADNP, ATF5, BARHL1, CEBPB, DBP, E2F7, ELF2, EVI1, FOXG1, GLI2, GLI3, HBP1, HIVEP1, HOXA13, HOXA1, HOXB13, HOXC6, LYL1, MED1, MNT, MYC, MZF1, NEUROG3, NFAT5, NFKB2, PATZ1, RAD21, RXRA, SA1, TAL1, TEAD2, TP73, ZNF143
9kb_contact_59	ELF2, HIVEP1, MED1, NFAT5, RAD21, RFX5, RXRA, TAL1, ZBTB10
9kb_contact_60	ADNP, HIVEP1, SMC3, ZBED4
9kb_contact_61	ADNP, HIVEP1, SMC3, ZBED4
9kb_contact_64	CUX1, E2F2, Ets-2, FOXG1, HOXB13, HOXC6, JUND, LYL1, MED12, MED1, MNT, MYC, NIPBL, RAD21, RXRA, SMC1A, SMC3, STAT2, TAL1, ZBED4, ZNF217
9kb_contact_65	E2F2, Ets-2, FOXG1, HOXA1, HOXA7, JUND, LYL1, MED12, MED1, MNT, MYC, NIPBL, RAD21, RXRA, SMC1A, SMC3, TAL1
9kb_contact_66	E2F2, Ets-2, FOXG1, HOXA1, HOXB13, HOXC6, JUN, JUND, LYL1, MED12, MED1, MNT, MYC, NEUROG3, NIPBL, RAD21, RXRA, SMC1A, SMC3, SREBF2, TAL1, TEAD2, ZBED4
9kb_contact_69	E2F3, ELF2, FOXP1, HOXA10, IRF1, JUND, MED12, MYC, RAD21, RXRA, SMC1A, SMC3
9kb_contact_70	RAD21, SMC1A, SMC3
9kb_contact_71	ADNP, E2F7, Ets-2, EVI1, HOXA13, JUND, MED12, NIPBL, PATZ1, RAD21, RFX5, RORA, SA1, SMC1A, SMC3, ZBED4, ZNF217,
9kb_contact_72	HIVEP1, RAD21
9kb_contact_73	ADNP, ATF1, ATF5, CEBPB, CUX1, DLX1, E2F2, E2F7, EGR1, EHF, ELF3, Ets-2, EVI1, FOXA2, FOXG1, GLI2, GMEB1, HIVEP1, HOXA13, HOXB13, JUND, LYL1, MED12, MED1, MNT, MYC, NFE2L2, NIPBL, PATZ1, RAD21, RXRA, SA1, SMC1A, SMC3, TAL1, VEZF1, ZBED4, ZNF217, ZNF250
9kb_contact_74	ADNP, ATF5, BARHL1, CEBPB, DBP, E2F7, EVI1, FOXG1, GLI2, GLI3, GMEB1, HBP1, HIVEP1, HOXA13, HOXB13, HOXC6, LYL1, MED1, MNT, MYC, MZF1, NFAT5, NFE2L2, PATZ1, RAD21, RARG, RFX5, RORA, RXRA, SA1, TAL1, VEZF1, ZBTB10, ZNF143, ZNF250
9kb_contact_75	ADNP, HIVEP1, SMC3, ZBED4
9kb_contact_76	CUX1, E2F2, Ets-2, FOXG1, HOXB13, HOXC6, JUND, LYL1, MED12, MED1, MNT, MYC, NIPBL, RAD21, RXRA, SMC1A, SMC3, STAT2, TAL1, ZBED4, ZNF217
9kb_contact_78	ATF1, LYL1, RXRA
9kb_contact_79	ATF1, ATF5, CEBPB, DBP, DLX1, E2F2, E2F7, EGR1, ELF3, Ets-2, EVI1, FOXG1, FOXP1, GLI2, HIVEP1, JUND, LYL1, MED12, MED1, MNT, MYC, NFAT5, NIPBL, PATZ1, RAD21, RORA, RXRA, SMC1A, SMC3, TAL1, TCF7L2, VEZF1, ZBTB10, ZNF143

9kb_contact_81	ADNP, E2F7, Ets-2, EVI1, RFX5, RORA, SMC3, ZBED4
9kb_contact_82	HIVEP1
9kb_contact_83	ADNP, ATF5, BARHL1, CEBPB, DBP, E2F7, EVI1, FOXG1, GMEB1, HIVEP1, HOXC6, LYL1, NFAT5, NFE2L2, RFX5, RORA, ZNF250
9kb_contact_84	ADNP, HIVEP1, SMC3, ZBED4
9kb_contact_85	NIPBL, RAD21, SMC1A, SMC3
9kb_contact_89	ATF1
9kb_contact_90	LYL1, RXRA
9kb_contact_92	ATF1, LYL1, RXRA
9kb_contact_93	RXRA
9kb_contact_95	ATF1, Ets-2, FOXP1, JUND, NIPBL, RAD21, SMC1A, SMC3
9kb_contact_96	BARX1, GLI2, JUND, NIPBL
9kb_contact_97	E2F7, Ets-2, EVI1, JUND, MED12, NIPBL, PATZ1, RAD21, RORA, SMC1A, SMC3
9kb_contact_98	ATF5, NIPBL, RAD21, SMC1A, SMC3
9kb_contact_99	HIVEP1, RAD21
9kb_contact_100	ATF1, ATF5, BARX1, CEBPB, DLX1, E2F2, E2F7, ELF3, Ets-2, EVI1, FOXG1, FOXP1, GLI2, GMEB2, HIVEP1, JUND, LYL1, MED12, MED1, MNT, MYC, NIPBL, PATZ1, RAD21, RXRA, SMC1A, SMC3, TAL1, TP73, VEZF1
9kb_contact_101	ATF1, ATF5, CEBPB, DLX1, E2F2, E2F7, EGR1, ELF3, Ets-2, EVI1, FOXG1, GLI2, GMEB2, HIVEP1, HOXA1, HOXA7, JAZF1, JUND, LYL1, MED12, MED1, MNT, MYC, NIPBL, PATZ1, RAD21, RXRA, SMC1A, SMC3, TAL1, TP73, VEZF1
9kb_contact_102	BARX1, DLX1, E2F7, Ets-2, EVI1, FOXP1, HIVEP1, JUND, LYL1, MED12, MED1, NIPBL, PATZ1, RAD21, RXRA, SMC1A, SMC3,
9kb_contact_103	ATF1, ATF5, BARX1, CEBPB, DLX1, E2F2, E2F7, EGR1, ELF3, Ets-2, EVI1, FOXG1, FOXP1, GMEB2, HIVEP1, HOXA1, HOXA7, JAZF1, JUND, LYL1, MED12, MED1, MNT, MYC, NFAT5, NIPBL, PATZ1, RAD21, RXRA, SMC1A, SMC3, TAL1, TP73, ZNF143
9kb_contact_104	ATF5, CEBPB, DBP, E2F7, EVI1, FOXG1, GLI2, HIVEP1, HOXA1, JAZF1, LYL1, MED1, MNT, MYC, NFAT5, PATZ1, RAD21, RORA, RXRA, TAL1, TP73, VEZF1, ZBTB10, ZNF143
9kb_contact_105	E2F2, Ets-2, FOXG1, HOXA1, HOXA7, JUND, LYL1, MED12, MED1, MNT, MYC, NIPBL, RAD21, RXRA, SMC1A, SMC3, TAL1
9kb_contact_106	ATF1, ATF5, CEBPB, DBP, DLX1, E2F2, E2F7, EGR1, ELF3, Ets-2, EVI1, FOXG1, FOXP1, GLI2, HIVEP1, JUND, LYL1, MED12, MED1, MNT, MYC, NFAT5, NIPBL, PATZ1, RAD21, RORA, RXRA, SMC1A, SMC3, TAL1, TCF7L2, VEZF1, ZBTB10, ZNF143
9kb_contact_107	ADNP, ATF1, CTCF, EHF, Ets-2, FOXP1, HOXC6, JUND, NIPBL, RAD21, SMC1A, SMC3
9kb_contact_108	ADNP, CTCF, E2F7, ERM, Ets-2, EVI1, HOXA13, HOXA6, JUN, JUND, MED12, MXD1, NIPBL, PATZ1, RAD21, REST, SA1, SMC1A, SMC3, STAT3, ZBED4
9kb_contact_109	ATF5, NIPBL, RAD21, SMC1A, SMC3
9kb_contact_110	HIVEP1, RAD21

9kb_contact_111	ADNP, ATF1, ATF5, CEBPB, CLOCK, DLX1, E2F2, E2F7, EGR1, EHF, ELF2, Ets-2, EVI1, FOXA2, FOXG1, GLI2, HIVEP1, HOXA13, HOXA1, HOXB13, JUN, JUND, LYL1, MED12, MED1, MNT, MYC, NIPBL, PATZ1, RAD21, RXRA, SA1, SMC1A, SMC3, TAL1, TEAD2, TP73, ZBED4
9kb_contact_112	ADNP, BARX1, CLOCK, DLX1, E2F7, Ets-2, EVI1, FOXA2, FOXO3, FOXP1, HBP1, HIVEP1, HOXB13, IRF1, JUND, LYL1, MED12, MED1, MXD1, NIPBL, PATZ1, RAD21, REST, RUNX1, RXRA, SMC1A, SMC3, TCF7L2
9kb_contact_113	ADNP, ATF5, BARHL1, CEBPB, DBP, E2F7, ELF2, EVI1, FOXG1, GLI2, GLI3, HBP1, HIVEP1, HOXA13, HOXA1, HOXB13, HOXC6, LYL1, MED1, MNT, MYC, MZF1, NEUROG3, NFAT5, NFKB2, PATZ1, RAD21, RXRA, SA1, TAL1, TEAD2, TP73, ZNF143
9kb_contact_114	E2F2, Ets-2, FOXG1, HOXA1, HOXB13, HOXC6, JUN, JUND, LYL1, MED12, MED1, MNT, MYC, NEUROG3, NIPBL, RAD21, RXRA, SMC1A, SMC3, SREBF2, TAL1, TEAD2, ZBED4
9kb_contact_115	ESRRA, LHX2, MED12, MYC, NIPBL, RAD21, RXRA, SMC1A, ZNF143
9kb_contact_116	Ets-2, EVI1, HOXA13, JUND, MED12, NIPBL, PATZ1, RAD21, RORA, SMC1A, SMC3
9kb_contact_118	ESRRA, LHX2, MED12, MYC, NIPBL, RAD21, RXRA, SMC1A, ZNF143
9kb_contact_119	CTCF, Ets-2, NIPBL, RAD21, SMC1A, SMC3
9kb_contact_120	CTCF, Ets-2, HOXC11, MED12, NIPBL, RAD21, RFX5, SMC1A, SMC3, ZBED4
9kb_contact_121	NIPBL, RAD21, SMC1A, SMC3
9kb_contact_123	HIVEP1, RAD21, SOX9
9kb_contact_124	CUX1, ELF2, Ets-2, FOXA2, HIVEP1, HNF4A, MED12, MED1, NIPBL, RAD21, RXRA, SMC1A, SMC3, TAL1, ZBED4
9kb_contact_125	Ets-2, FOXA2, HIVEP1, HNF4A, HOXC11, MED12, MED1, NIPBL, RAD21, RXRA, SMC1A, SMC3
9kb_contact_126	ELF2, HIVEP1, MED1, NFAT5, RAD21, RFX5, RXRA, TAL1, ZBTB10
9kb_contact_128	RXRA
9kb_contact_129	H3, HINFP
9kb_contact_134	ATOH1, H3, HES1, ZBTB10
9kb_contact_135	EVI1, NR3C1, RAD21, SMC1A
9kb_contact_146	H3, HINFP

Supplementary Methods

Supplementary Equation 1

At iteration l:

$$\mathcal{M}_{ij}^{(l+1)} = \frac{\mathcal{M}_{ij}^{(l)}}{\frac{\frac{w_i}{\mathcal{N}_i}}{\frac{\mathcal{W}_i}{\mathcal{A}_i}} \cdot \frac{\frac{w_j}{\mathcal{N}_j}}{\frac{\mathcal{W}_j}{\mathcal{A}_j}}} = \frac{\mathcal{M}_{ij}^{(l)}}{\tilde{w}_i \cdot \tilde{w}_j}$$

where:

$$\begin{aligned}
w_i &= \sum_{j \in T_i} \mathcal{M}_{ij}^{(l)} \\
\mathcal{N}_i &= \sum_{j \in T_i} \mathbf{1}(\mathcal{M}_{ij}^{(l)} > 0) \\
\mathcal{W}_i &= \sum_{i \in S_i} w_i \\
\mathcal{A}_i &= \sum_{i \in S_i} \mathcal{N}_i \\
T_i &= \{k : \text{ bin } k \text{ is enriched and on a different chromosome than bin } i\} \\
S_i &= \begin{cases} \{k : \text{ bin } k \text{ is enriched}\}, & \text{if bin } i \text{ is enriched} \\ \{k : \text{ bin } k \text{ is non-enriched}\}, & \text{if bin } i \text{ is non-enriched} \end{cases} \\
\mathbf{1}(x) &= \begin{cases} 1, & \text{if } x \text{ is true} \\ 0, & \text{otherwise} \end{cases}
\end{aligned}$$

4C-seq

4C-seq libraries were prepared as per previously described by van de Werken *et al*[2]. Briefly, 10 million Colo205, LoVo and LS174T cells were cross-linked with 2% formaldehyde and lysed. We used *Hind*III as the primary restriction enzyme (RE) and the 4bp cutter (*Dpn*II, *Csp6*I or *Nla*III) as the secondary RE (fragend >300bp). 4C viewpoints chosen from the cHi-C data for validation were >1,500bp. Primers were designed using the 4C primer design software (Supplementary Table 8). Reading and non-reading primers incorporated P7 and P5 Illumina single end adapter sequence as 5' overhangs, respectively. Multiplexed 4C libraries for each cell-line were sequenced on an Illumina

HiSeq2000 instrument (one lane per cell-line) to obtain 100bp single -end reads. 35-40% PhiX library was added to each lane to enhance sequence diversity.

Reads were parsed based on unique viewpoint specific primer sequences. Reads were then mapped to the human genome (hg19) using Bowtie (version 2.1.0). The raw experimental data included 740,000-11,000,000 reads per viewpoint. Using Bowtie2[3], we uniquely aligned on average 72% of these reads. We initially filtered reads according to PHRED score as in our cHi-C analysis. Reads that passed quality filtering were considered to represent genuine ligation events between the viewpoint fragment and the other primary restriction-fragment end. Implementing standard procedures[2], we allocated unique 4C-seq reads to fragments (*i.e.* blind and non-blind fragments) using an in-house pipeline. Profiles for the two classes of fragments were obtained at 100bp resolution and an average profile for a 5Kb running window was computed. 4C-seq data profiles were distance normalized as for the cHi-C data.

Fluorescence *in-situ* hybridisation (FISH)

Analysis of co-localization frequencies using interphase FISH as shown by Simonis *et al*[4] can be used to validate long-range interactions. Relevant cHi-C interactions should co-localize at significantly higher frequency than random control probes at similar distance. We designed FISH probe sets for testing far-*cis* (>5Mb) and trans interactions which comprise a SNP having an association *p*-value within one order of magnitude with the region's top associated SNP. One probe set consists of three probes covering test bin (Probe_1), interactor bin (Probe_2), and a control region (Probe_3) picked randomly 5Mb upstream or downstream of Probe_2 (Supplementary Table 3). Probes were generated from bacterial artificial chromosome (BAC) clones (BACPAC Resources Centre, Oakland, CA, USA) listed in Supplementary Table 3 as previously described [5]. Briefly, BAC DNA (2ug) was labelled by nick translation (Probe_1: Green-dUTP, Probe_3: Aqua-dUTP (both Abbott Molecular, Des Plaines, IL, USA); Probe_2: Cy3-dUTP (GE Healthcare, Little Chalfont, UK)), purified using MicroSpin G-50 columns (GE Healthcare, Piscataway, NJ, USA), and ethanol precipitated with 50µg salmon sperm DNA (Sigma, St. Louis, MO) and 50µg E. coli tRNA (Roche, Basel, Switzerland). Per set, 3ul of each probe were precipitated with 15µg of Cot-1, resuspended in 11µl of SSPE-based hybridization buffer and applied to cells previously fixed onto a microscope slide sequentially applying 0.075M KCl (37°C, 10min) and a 3:1 methanol to glacial acetic acid solution (4°C, 30min). Hybridization was performed over-night at 37°C and slides were processed using the Vysis wash protocol[5]. Nuclei were stained with DAPI contained in VectaShield Mounting Medium (Vector Laboratories, Burlingame, CA, USA). Metaphase slides were viewed on a Zeiss Axioskop epifluorescence microscope equipped with filters matching the fluorochromes used. Images were captured and fluorescence signals analyzed using a charge-coupled device (Photometrics, Tucson, AZ) and SmartCapture software

(Digital Scientific, Cambridge, UK). Interphase slides were scanned using a Bioview Duet-3 station (microscope: Olympus bx61; camera: u-cmad3; software: duet 3.4.0.21) at 60-fold magnification.

Specific hybridization of probes to the correct location was confirmed on normal Metaphases derived from peripheral blood mononuclear cells, and non-specific probes were omitted from subsequent interphase FISH in LS174T cells (Supplementary Table 3). Three circular spots (1mm diameter) on interphase FISH slides were scanned, capturing up to 4000 nuclei per probe set. Image analysis was performed in an automated, agnostic fashion. The location of each nucleus on the scanned tile and the coordinates of FISH probes were extracted using a Fiji[6] macro. A binary mask was computed by executing the following Fiji functions: (i) Background substraction (ii) Median filtering, (iii) Thresholding, (iv) Fill holes, (v) Morphological erode, (vi) Analyze Particles and a subsequent watershed *trans*formation. The FISH probes were detected by employing the following macro: (i) Background substraction, (ii) Gaussian blurring and a subsequent (iii) entropy thresholding. After image pre-processing, tiles were analyzed using the statistical computation package R[7].

Quality of *in silico* processing steps was visually evaluated by an expert (AH) blinded for the probe set design. After *in silico* quality filtering, we required the presence of at least 200 high-quality nuclei (*i.e.* n=400 alleles) for proceeding to co-localization analysis. Fisher's exact test requires n>400 to have >90% power in declaring a representative difference in co-localization frequencies (chosen here to be 5% and 1%, respectively) significant at p<0.05. Distances between all probes were measured from the closest peripheral pixel, and co-localization was defined as a distance of zero pixels or less (signal overlap) (Figure 5). The two alleles per nucleus were scored under the assumption that co-localisation of *cis* probe sets represent intra-chromosomal contacts, in line with 3C technologies where *trans* interactions between homologous chromosomes cannot be distinguished from *cis* interactions. Percentages of co-localising alleles were calculated and a Fisher's exact test was used to assign a p-value to the observed difference. For three slides, >100 nuclei each were counted manually, and observed co-localisation frequencies were consistent with results from the computational image analysis.

Supplementary References

1. Imakaev M*, et al.* Iterative correction of Hi-C data reveals hallmarks of chromosome organization. *Nat Methods* **9**, 999-1003 (2012).

2. van de Werken HJ*, et al.* Robust 4C-seq data analysis to screen for regulatory DNA interactions. *Nature methods* **9**, 969-972 (2012).

3. Langmead B, Salzberg SL. Fast gapped-read alignment with Bowtie 2. *Nature methods* **9**, 357-359 (2012).

4. Simonis M*, et al.* Nuclear organization of active and inactive chromatin domains uncovered by chromosome conformation capture-on-chip (4C). *Nat Genet* **38**, 1348-1354 (2006).

5. Buckle V, Rack K. Fluorescent in situ hybridisation. *In: Davies KE, editor Human Genetic Analysis Oxford: IRL Press*, pp. 59–82. (1993).

6. Schindelin J*, et al.* Fiji: an open-source platform for biological-image analysis. *Nat Methods* **9**, 676-682 (2012).

7. R Core Team. *R: A language and environment for statistical computing*. Foundation for Statistical Computing (2013).
